# Osteopontin Expression Identifies a Subset of Recruited Macrophages Distinct from Kupffer Cells in the Fatty Liver

**DOI:** 10.1016/j.immuni.2020.08.004

**Published:** 2020-09-15

**Authors:** Anneleen Remmerie, Liesbet Martens, Tinne Thoné, Angela Castoldi, Ruth Seurinck, Benjamin Pavie, Joris Roels, Bavo Vanneste, Sofie De Prijck, Mathias Vanhockerhout, Mushida Binte Abdul Latib, Lindsey Devisscher, Anne Hoorens, Johnny Bonnardel, Niels Vandamme, Anna Kremer, Peter Borghgraef, Hans Van Vlierberghe, Saskia Lippens, Edward Pearce, Yvan Saeys, Charlotte L. Scott

**Affiliations:** 1 Laboratory of Myeloid Cell Biology in Tissue Damage and Inflammation, VIB-UGent Center for Inflammation Research, Technologiepark-Zwijnaarde 71, Ghent 9052, Belgium; 2 Department of Biomedical Molecular Biology, Faculty of Science, Ghent University, Ghent, Belgium; 3 Laboratory of Myeloid Cell Biology in Tissue Homeostasis and Regeneration, VIB-UGent Center for Inflammation Research, Technologiepark-Zwijnaarde 71, Ghent 9052, Belgium; 4 Max Planck Institute of Immunobiology and Epigenetics, Freiburg, Germany; 5 Data Mining and Modelling for Biomedicine, VIB-UGent Center for Inflammation Research, Technologiepark-Zwijnaarde 71, Ghent 9052, Belgium; 6 Department of Applied Mathematics, Computer Science and Statistics, Faculty of Science, Ghent University, Ghent, Belgium; 7 VIB BioImaging Core, VIB-UGent Center for Inflammation Research, Technologiepark-Zwijnaarde 71, Ghent 9052, Belgium; 8 Department of Basic and Applied Medical Sciences, Faculty of Medicine and Health Sciences, Ghent University, Belgium; 9 Department of Pathology, Ghent University Hospital, Ghent 9000, Belgium; 10 Department of Gastroenterology and Hepatology, Ghent University Hospital, Ghent 9000, Belgium; 11 University of Freiburg, Freiburg, Germany

**Keywords:** macrophages, MAFLD, NAFLD, lipid, subsets, Kupffer cells, liver, NASH, Western Diet

## Abstract

Metabolic-associated fatty liver disease (MAFLD) represents a spectrum of disease states ranging from simple steatosis to non-alcoholic steatohepatitis (NASH). Hepatic macrophages, specifically Kupffer cells (KCs), are suggested to play important roles in the pathogenesis of MAFLD through their activation, although the exact roles played by these cells remain unclear. Here, we demonstrated that KCs were reduced in MAFLD being replaced by macrophages originating from the bone marrow. Recruited macrophages existed in two subsets with distinct activation states, either closely resembling homeostatic KCs or lipid-associated macrophages (LAMs) from obese adipose tissue. Hepatic LAMs expressed Osteopontin, a biomarker for patients with NASH, linked with the development of fibrosis. Fitting with this, LAMs were found in regions of the liver with reduced numbers of KCs, characterized by increased Desmin expression. Together, our data highlight considerable heterogeneity within the macrophage pool and suggest a need for more specific macrophage targeting strategies in MAFLD.

## Highlights

• Resident KCs are lost with time in MAFLD
• Resident KCs are replaced by distinct subsets of bone marrow derived macrophages
• One subset of recruited macrophages termed hepatic LAMs, express Osteopontin
• Hepatic LAMs are found in zones characterized by increased Desmin expression

## Graphical Abstract

**Figure:**
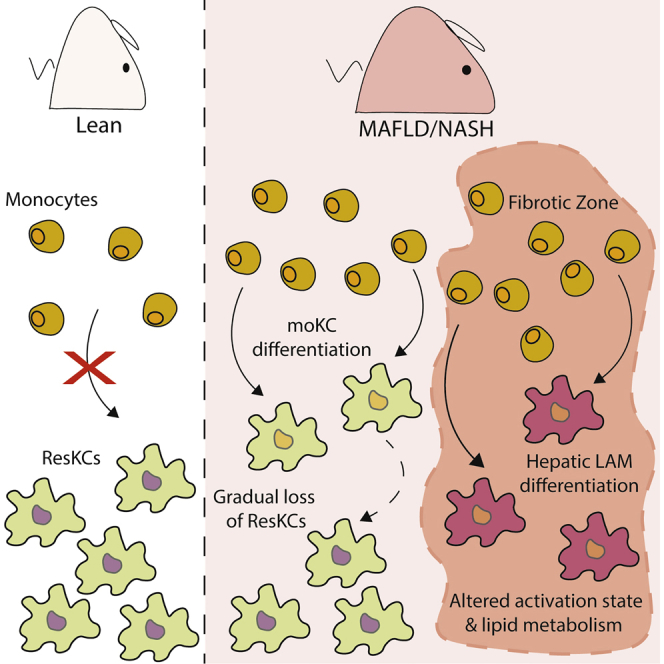

## Teaser

Hepatic macrophages are thought to play key roles in the pathogenesis of fatty liver disease; however, heterogeneity within the macrophage pool remains largely unstudied. In this issue of *Immunity*, Remmerie et al. report a population of osteopontin-expressing macrophages with a unique transcriptional profile and location in the fatty liver.

## Introduction

Due to the increasing prevalence of obesity and associated insulin resistance, non-alcoholic fatty liver disease (NAFLD), recently renamed metabolic associated fatty liver disease (MAFLD) (Eslam et al., 2020), has become a global problem (Byrne and Targher, 2015). MAFLD consists of a spectrum of disease states ranging from simple steatosis to the more end-stage of the disease termed non-alcoholic steatohepatitis (NASH), encompassing fibrosis, cirrhosis and hepatocellular carcinoma (HCC). Not all patients progress from the steatosis phase to NASH, and the reasons underlying the progression remain unclear. Additionally, as there is currently no treatment for NASH, liver transplantation is often the only option. Thus, further research is required to understand MAFLD pathogenesis and design new treatment strategies.

It is commonly accepted that multiple hits coming from the gut and adipose tissue are key regulators of disease progression (Buzzetti et al., 2016). Hepatic macrophages (macs) have been implicated in this process, being activated to become pro-inflammatory by the excess lipids and damage in the fatty liver and by signals originating from the intestine. However, it is not clear which macs are involved. Kupffer cells (KCs) are the main mac population in the healthy liver, where they reside with at least part of their body in the liver sinusoids, interacting with liver sinusoidal endothelial cells (LSECs), hepatic stellate cells (HSCs), and hepatocytes (Bonnardel et al., 2019). Although there has been considerable interest in the role of KCs in MAFLD, many existing studies have relied on the use of non-specific markers to identify “KCs,” together with non-specific methods like clodronate-liposome-mediated depletion to study their functions. It has recently become clear that these approaches cannot distinguish between resident KCs (ResKCs) and other macs, recruited to the liver in response to inflammation. As a result, these poorly defined “KCs” have been attributed many, often contradictory, roles in MAFLD, and the relative contributions of bona fide KCs and recruited macs remain largely unknown (Remmerie and Scott, 2018). By developing tools to identify and manipulate KCs based on their expression of the C-type lectin, CLEC4F, we have shown that ResKCs are replaced by monocyte-derived KCs (moKCs) upon depletion with diphtheria toxin and that with time, these moKCs then become resident (Scott et al., 2016). We have previously reported a similar replacement of the ResKC pool by moKCs in mice fed a methionine- and choline-deficient diet (MCD), a protocol that leads to a NASH-like disorder (Devisscher et al., 2017). However, whether this holds true in a more clinically relevant model of MAFLD remains to be seen.

Here, we have employed cellular indexing of transcriptomes and epitopes by sequencing (CITE-seq) to investigate the transcriptomes and surface epitopes of CD45^+^ cells in the livers of mice with MAFLD, induced by feeding a Western diet (WD) for 12, 24, or 36 weeks. In combination with flow cytometry, fate mapping, and confocal microscopy, this revealed that ResKCs are not pro-inflammatory in MAFLD. Rather, ResKCs are gradually lost as the disease progresses, being replaced by moKCs. Monocytes recruited to the liver also differentiated into a distinct subset of osteopontin-expressing CLEC4F^-^ macs with a transcriptome similar to that of lipid-associated macs (LAMs) in adipose tissue (Jaitin et al., 2019) and scar-associated macs in the fibrotic human liver (Ramachandran et al., 2019) and hence were termed hepatic LAMs. Notably, hepatic LAMs were differentially activated as compared with ResKCs and moKCs and had distinct abilities to metabolize lipids. Together, our data reveal considerable heterogeneity within the hepatic macrophage pool in MAFLD and suggest a need for more specific mac targeting strategies.

## Results

### MAFLD Induces Changes in the Transcriptome and Surface Proteome of Hepatic Immune Cells

To assess the roles of hepatic macs in MAFLD, mice were fed a Western diet (WD) consisting of excess fat and cholesterol and supplemented with sucrose and fructose in the drinking water, a protocol generating all stages of human MAFLD and NASH (Ganz et al., 2015). Control mice were fed a standard diet (SD). WD-fed mice gained a significant amount of weight compared with SD-fed mice (Figure S1A) and had an increased liver weight and liver to body weight ratio (Figure S1B). From 12 weeks, WD-fed mice also showed progressively elevated serum AST and ALT, cholesterol, triglyceride and fasting insulin concentrations as well as glucose intolerance (Figures S1C–S1F). Histological analysis confirmed that WD-fed mice exhibited all features of human MAFLD, with NASH developing at later time points (Caldwell et al., 2010; Sethunath et al., 2018) (Figures S1G and S1H), while some of these mice (1/82 at 12 weeks, 2/77 at 24 weeks, and 5/34 at 36 weeks) also developed HCC.

**Figure 1:**
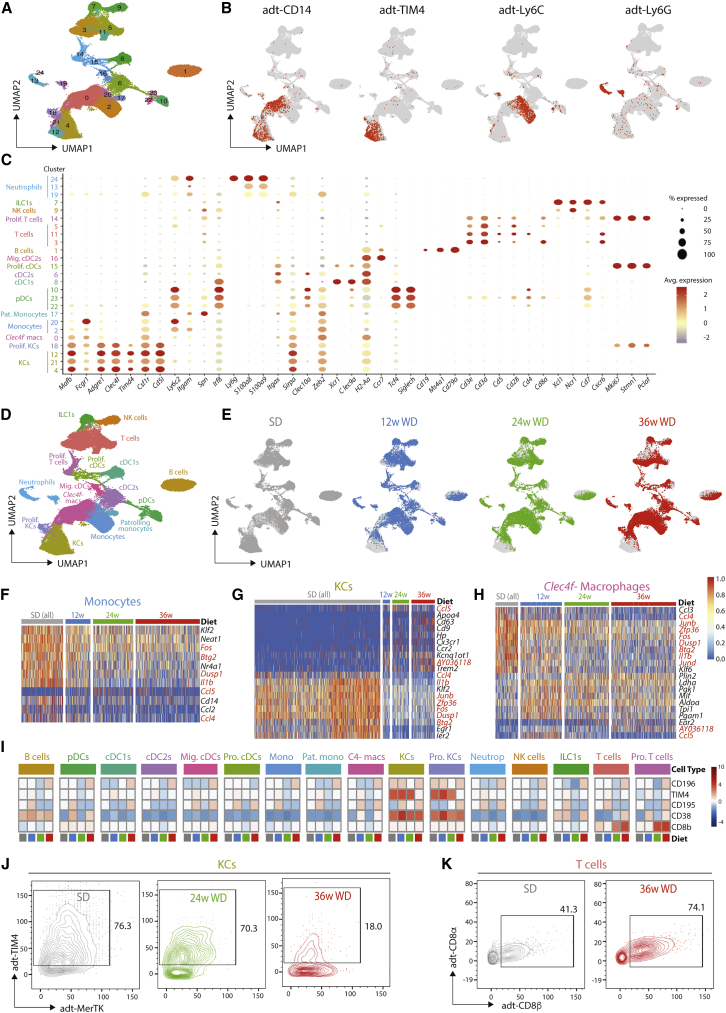
Hepatic Immune Cell Transcriptome and Surface Proteome in MAFLD C57BL/6 mice were fed either an SD or WD for 12, 24, or 36 weeks, and livers were harvested. Total live CD45^+^ cells were sorted (1 mouse per time point per diet), stained with total-seq A antibodies, and loaded onto the 10X Chromium platform. After QC, 56407 cells remained. (A) UMAP showing distinct clusters among total CD45^+^ live cells. (B) Expression of indicated proteins based on CITE-Seq antibody binding. (C) Expression of indicated genes across the 25 clusters. (D) Annotation of the cell types within the UMAP based on both transcriptome and surface proteome. (E) Distribution of clusters from SD or WD, with SD data obtained from cells pooled after 12, 24, and 36 weeks. (F–H) Heatmaps showing top DEGs for Monocytes (F), KCs (G), and *Clec4f*^-^ Macrophages (H) as assessed by comparing SD and WD samples pooled from all time points. Genes in red are conserved across multiple cell types. (I) MEM heatmap showing surface proteins whose expression was altered in at least 1 cell type during MAFLD. (J and K) CITE-Seq data were exported into FlowJo software, and (J) the KC cluster was gated and TIM4 and MerTK expression were examined at indicated time points on WD and in pooled SD-fed mice or (K) the T cell cluster was gated and CD8α and CD8β expression were examined at 36 weeks on WD and in pooled SD-fed mice. See also Figure S2.

To investigate mac heterogeneity in MAFLD, we employed CITE-Seq (Peterson et al., 2017; Stoeckius et al., 2017). To avoid bias, total live CD45^+^ cells from the liver were isolated from a total of 6 mice (one from each of the SD- and WD-fed groups at 12, 24, or 36 weeks), stained with a panel of 112 antibodies including isotype controls, and loaded onto the 10X Genomics Chromium platform. After sequencing, aggregation of the samples, quality control, removal of contaminating CD45^-^ cells, and exclusion of cells resembling doublets, a total of 56,407 cells remained (6,116 cells from 12-week SD, 10,465 cells from 12-week WD, 5,357 from 24-week SD, 10,788 from 24-week WD, 11,018 cells from 36-week SD, and 12,663 from 36-week WD). 25 clusters could be identified by generating a UMAP from the transcriptome data using 20 principal components (Figure 1A). 16 discrete cell types could be identified based on the differentially expressed genes (DEGs) (Figure S2A, Table S1, www.livercellatlas.org), combined with protein expression profiles (Figures 1B, S2B, and S2C) and their expression of a set of standard cell identity genes (Figure 1C), including B cells, T cells, NK cells, patrolling monocytes, classical monocytes, KCs (*Clec4f*^+^), other macrophages (*Clec4f*^*-*^*)*, pDCs, cDC1s, and cDC2s (Figure 1D). Three additional clusters (13, 19, and 24) were designated as neutrophils based on their protein expression of Ly6G (Figure 1B). However, only one of these (cluster 24) expressed mRNA for *Ly6g* (Figure 1C), likely reflecting the technical difficulties involved in isolating good quality mRNA from neutrophils.

Examination of the distribution of cells originating from the mice fed the different diets at each time point suggested that many cells, including macs, B cells, and cDC1s, were altered in MAFLD (Figure 1E). To investigate this further, we assessed how the transcriptome of each population was affected. This effort found multiple cell types with a set of conserved DEGs (Figures 1F–1H and S2D; Table S1), including increased *Ccl5* expression, which has previously been associated with steatosis and fibrosis (Berres et al., 2010; Kim et al., 2018; Kirovski et al., 2010) and decreased expression of *Ccl4* and the TIS11 family members, *Zfp36*, *Zfp36l1*, and *Zfp36l2*, thought to function in post-transcriptional gene degradation (Baou et al., 2009) (Figures 1F–1H and S2D). A number of DEGs were also cell-type specific (Figures 1F–1H and S2D; Table S1). CITE-Seq analysis found 5 surface proteins to be differentially expressed by one or more cell types over time in MAFLD, including increased expression of CD8β by T cells and reduced TIM4 on KCs (Figures 1I–1K). The increase in CD8^+^ T cells is in agreement with recent findings in mice and humans (Bhattacharjee et al., 2017; Ghazarian et al., 2017; Haas et al., 2019), highlighting the robustness of our approach. Based on our previous work (Devisscher et al., 2017; Scott et al., 2016), the decrease in TIM4 expression in KCs suggests that ResKCs may be replaced by newly recruited macs.

### Hepatic Myeloid Cells Are Heterogeneous in MAFLD

**Figure 2:**
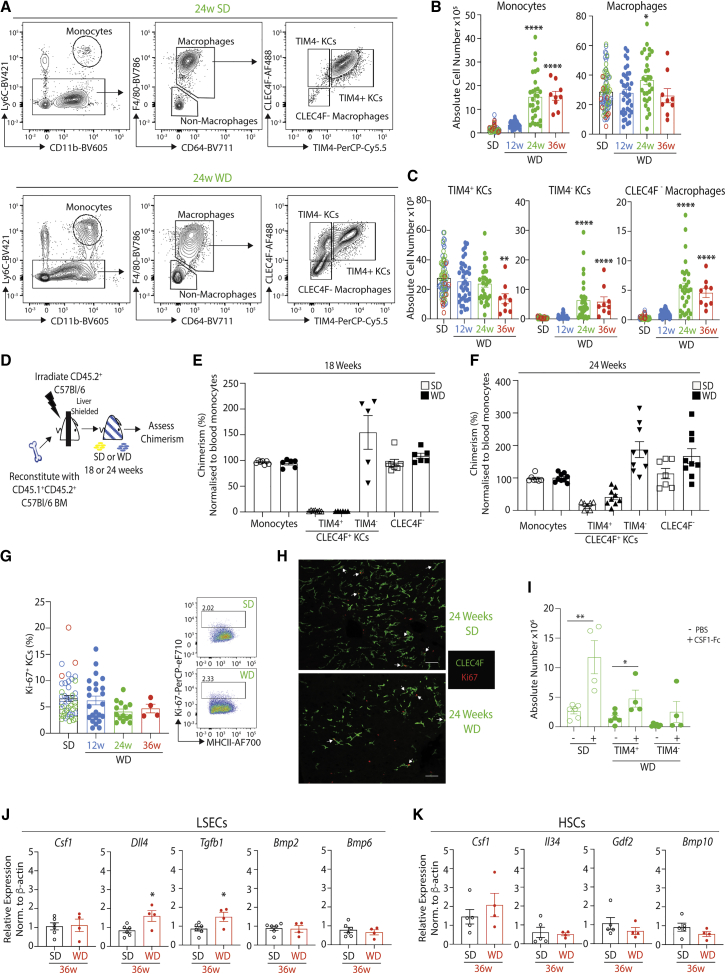
Loss of TIM4^+^ Resident KCs and Replacement from the BM in MAFLD (A) Gating strategy used to identify monocyte and mac populations in all figures (for full gating strategy, see Figure S3A). (B and C) Absolute cell numbers per liver of indicated cell types from mice fed the diets for 12 (blue), 24 (green), or 36 (red) weeks, excluding mice that developed HCC. Data are pooled from 3–7 independent experiments with n = 9–38. ^∗∗^p < 0.01, ^∗∗∗^p < 0.001, ^∗∗∗∗^p < 0.0001. One-way ANOVA compared with pooled SD. (D) Schematic showing generation of protected chimeras. (E and F) % chimerism (compared with blood monocytes) in indicated hepatic populations was assessed in protected chimeras 18 (E) or 24 (F) weeks after feeding the SD or WD. Data are pooled from 2 independent experiments with n = 5–9 per group. (G) % of Ki-67^+^ cells among Clec4F^+^ KCs in mice fed the WD for indicated time points, together with pooled results from SD-fed mice (left panel) and representative flow cytometry plots show Ki67 expression by Clec4F^+^ KCs from a mouse fed SD or WD for 24 weeks (right panels). Data are from 1 (36 weeks) or pooled from 3 (12 and 24 weeks) independent experiments with n = 3–24. (H) Confocal microscopy of livers of SD or WD-fed mice (24 weeks), showing expression of Clec4F (green) and Ki-67 (red). White arrows indicate Ki-67^+^ KCs. Images are representative of 2 mice per diet. (I) Absolute number of CLEC4F^+^TIM4^+^ ResKCs and CLEC4F^+^TIM4^-^ moKCs in SD- or WD-fed (24 weeks) mice injected with 1 mg/kg CSF1-Fc or PBS subcutaneously for 4 days before being sacrificed at day 6. Data are pooled from 2 independent experiments, with n = 4–6 per group. ^∗^p < 0.05, ^∗∗^p < 0.01, unpaired Student’s t test. (J and K) qPCR analysis for indicated genes in LSECs (J) and HSCs (K) sorted from SD- (black) or WD-fed (red) mice (36 weeks). Data are from 1 experiment, with n = 4–6 per group. ^∗^p < 0.05, Student’s t test. All error bars indicate SEM. See also Figure S3.

We next used flow cytometry to explore the macs present in the liver at the different time points of MAFLD (gating strategy in Figure S3A). This found an increase in total Ly6C^hi^ monocytes from 24 weeks on the WD compared with SD-fed mice (Figures 2A and 2B). Although only a slight increase in the number of macs defined as CD64^+^F4/80^+^ was observed at 24 weeks (Figures 2A and 2B), the numbers and proportions of CLEC4F^+^TIM4^-^ KCs and CLEC4F^-^ macs increased from 24 weeks on the WD. There was a concomitant reduction in the proportion of CLEC4F^+^TIM4^+^ ResKCs after feeding the WD for 24 weeks, and the number of these cells was reduced at 36 weeks (Figures 2A, 2C, and S3B). Notably, F4/80 and CD11b expression could not discriminate between the different macs (Figure S3C). In the few mice with HCC, CLEC4F^+^TIM4^+^ KCs were almost absent (Figures S3D and S3E). Increases in neutrophils, eosinophils, cDC1s, and cDC2s from 24 weeks on the WD (Figures S3F–S3H) were also observed, consistent with the histological evidence of inflammation (Figure S1G). While the total numbers of cDC1s and cDC2s were increased in WD-fed mice (Figure S3F), there was a reduction in the % of cDC1s and a reciprocal increase in the % of cDC2s (Figure S3G), consistent with a recent report (Haas et al., 2019). Moreover, as recently reported (Brown et al., 2019), hepatic cDC2s were heterogeneous in terms of Mgl1, CCR2, and Tbet expression (Figure S3H).

### CLEC4F^-^ Macs and TIM4^-^ KCs Are Recruited from the Bone Marrow

To test the hypothesis that CLEC4F^+^TIM4^-^ KCs and CLEC4F^-^ macs were newly recruited from the bone marrow (BM) in MAFLD, we generated shielded chimeras in which CD45.2 mice were irradiated with their livers protected before being reconstituted with congenic donor BM and put on the SD or WD (Figure 2D). Chimerism of the hepatic monocyte and mac populations was then compared with that of blood monocytes, which derive exclusively from the BM. 18 weeks after reconstitution, there was no chimerism of CLEC4F^+^TIM4^+^ ResKCs in SD- or WD-fed mice, whereas CLEC4F^+^TIM4^-^ KCs and CLEC4F^-^ macs were chimeric, demonstrating their BM origin (Figure 2E). As TIM4 has been described to be expressed with time (Bain et al., 2016; Scott et al., 2016; Shaw et al., 2018), to determine if the lack of chimerism in WD-fed ResKCs was because the CLEC4F^+^TIM4^-^ moKCs had not been in the tissue long enough to acquire TIM4 expression, we also analyzed chimerism after 24 weeks. A small proportion of CLEC4F^+^TIM4^+^ ResKCs were chimeric at this time, suggesting that while some moKCs may give rise to these cells, this is likely an inefficient process (Figure 2F). Notably, local proliferation of KCs was not found to contribute to the maintenance of their numbers in MAFLD, as the number of Ki67^+^ CLEC4F^+^ KCs was identical in SD- and WD-fed mice (Figures 2G and 2H). This was not due to an intrinsic inability of the ResKCs to proliferate in the diseased liver, as administration of CSF1-Fc to SD- or WD-fed mice (24 weeks) induced proliferation of TIM4^+^ ResKCs irrespective of the diet (Figure 2I). To determine if loss of signals originating from LSECs or HSCs recently described to drive KC differentiation and maintenance (Bonnardel et al., 2019; Sakai et al., 2019) were responsible for the reduced ResKC population, we examined these by qPCR (sorted as described in Bonnardel et al., 2019) after feeding the diets for 36 weeks. However, none of the LSEC- or HSC-derived signals were found to be reduced (Figures 2J and 2K); thus, loss of these signals was not driving loss of ResKCs.

### ResKC Niche Cells Undergo Increased Proliferation

**Figure 3:**
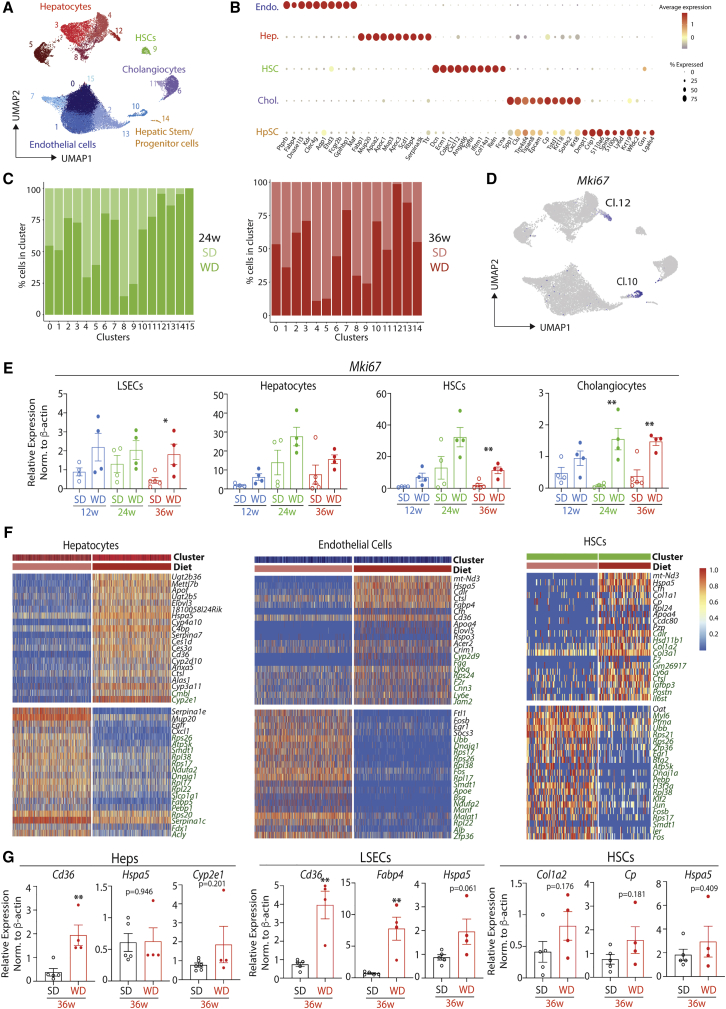
Changes in Hepatic Structural Cells in MAFLD C57BL/6 mice were fed an SD or WD for 24 or 36 weeks, and livers were harvested. Live CD45^-^ cells were then sorted (1 mouse per time point per diet) and loaded onto the 10X Chromium platform. After QC, 33,241 cells remained. (A) UMAP showing distinct clusters among total CD45^-^ live cells. (B) Expression of indicated genes across the 5 cell types. (C) Distribution of clusters from SD- or WD-fed mice at 24 weeks (green) or 36 weeks (red). (D) Expression of *Mki67* across the different clusters. (E) Expression of *Mki67* as determined by qPCR on indicated cells sorted from livers of SD- or WD-fed mice (12, 24, and 36 weeks). Data are from a single experiment, with n = 4–6 per group. ^∗^p < 0.05, ^∗∗^p < 0.01, Student’s t test. Error bars indicate ±SEM. (F) Heatmaps showing top 40 DEGs in the indicated cells types between SD- and WD-fed mice (24 and 36 weeks). Genes in green represent DEGs specifically altered at the 36-week time point. (G) qPCR analysis for indicated genes in indicated cell populations. Data are from a single experiment, with n = 4–5 per group. ^∗^p < 0.05, ^∗∗^p < 0.01. Error bars indicate ±SEM. See also Figure S4.

To further investigate the response of the ResKC niche cells to MAFLD, we next performed scRNA-seq analysis of live CD45^-^ cells from mice fed the SD or WD for 24 and 36 weeks (4 samples in total). After sequencing, aggregation of the samples, quality control, removal of ambient mRNA signals, alignment with the Harmony Algorithm (Korsunsky et al., 2019), removal of contaminating CD45^+^ cells, and exclusion of cells resembling doublets, a total of 33,241 cells remained (8,747 from 24-week SD, 11,759 from 24-week WD, 7,247 cells from 36-week SD, and 5,488 from 36-week WD). 15 clusters could be identified by generating a UMAP using 20 principal components (Figure 3A, www.livercellatlas.org). 5 discrete cell types, hepatocytes, endothelial cells, HSCs, cholangiocytes, and a small population of cells resembling hepatic stem or progenitor cells (HpSCs), could be identified by analysis of DEGs (Figure 3B; Table S2). Analysis of the origins of the cells found that some clusters were predominantly composed of cells originating from the WD-fed mice, including clusters 10 and 12 (Figure 3C). Closer inspection found that these clusters were proliferating endothelial cells and hepatocytes respectively (Figure 3D). qPCR for *Mki67* expression on purified cells from mice fed the diets for 36 weeks confirmed this increase in proliferation in LSECs and also identified increased proliferation in HSCs and Cholangiocytes (Figure 2E). A trend toward increased proliferation was also observed in the hepatocytes. Confocal microscopy for Ki-67 in mice fed either diet for 36 weeks further confirmed an increase in proliferation in MAFLD with Ki-67^+^ cells (Figure S4A).

In addition to increased proliferation, we also investigated other changes in the transcriptomes of the niche cells. To account for the heterogeneity between clusters of the same cell type (primarily due to zonation in hepatocytes and zonation and distinct endothelial cell subsets in endothelial cells) (Halpern et al., 2018; 2017) (Figures S4B–S4D), we examined DEGs between the different clusters from mice fed the SD or WD for 24 or 36 weeks. This identified a number of conserved DEGs between the clusters (Figures 3F and S4E; Table S3). Some of these were found after feeding the WD for 24 and 36 weeks, while some were specific to the mice fed the WD for 36 weeks (Figures 3F and S4E). Any DEGs moving in different directions at 24 and 36 weeks were excluded. Two conserved downregulated genes in the niche cells (*Fos* & *Zfp36*) were also observed in the CD45^+^ cells (Figures 1F–1H and S2D); however, the relevance of this remains to be examined. Some of the DEGs were confirmed using qPCR (Figure 3G); this included *Cd36* and *Fabp4*, indictive of the increased lipid load. Conversely, we were not able to validate some of the DEGs shown in the scRNA-seq analysis, such as *Hspa5* (Figure 3G). Moreover, confocal microscopy for HSPA5 also did not reveal any differences in protein expression (Figure S4G); however, as this primarily stained the hepatocytes, this analysis demonstrated the increased size of hepatocytes in WD- compared with SD-fed mice (Figure S4G). These results highlight that while the expression of signals important for ResKC development and maintenance are not lost in MAFLD, the niche cells have an altered transcriptome and also undergo increased proliferation in MAFLD. Additionally, hepatocytes increase in size which could alter the distribution of signals to ResKCs. These alterations to the niche could therefore play a role in the loss of ResKCs in MAFLD.

### Recruited Macrophages Colonize the ResKC Niche

**Figure 4:**
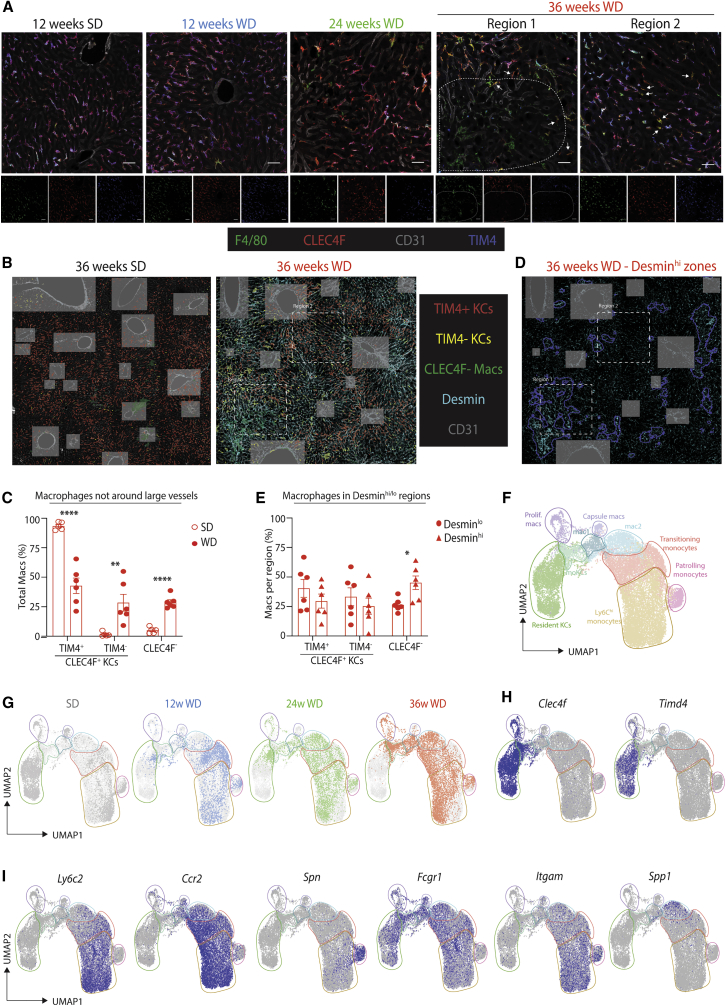
Localization and Heterogeneity of Macrophages in MAFLD (A) Confocal microscopy showing cells expressing CLEC4F (red), F4/80 (green), TIM4 (blue), and CD31 (gray) in the livers of SD- or WD-fed mice at the indicated time points. Smaller images show results for individual channels; the larger image is merged from all channels. Scale bar, 50 μm. Images are representative of 5–6 mice per time point and are extracted from 4 × 4 tiled images. White arrows point to CLEC4F^+^TIM4^-^ moKCs. Dashed line highlights the zones enriched for CLEC4F^-^ macs. (B) Tile scans (4x4) of livers from SD- and WD-fed mice (36 weeks) showing annotation of identified macs per subset in indicated colors. Indicated regions (dashed lines) identify the areas from the WD-fed mouse used in (A). Shaded gray boxes identify large vessels (portal or central veins) that were excluded from quantification analysis in (C) and (E). Images are representative of 6 mice. (C) Quantification of indicated populations shown in (B) as a % of total F4/80^+^ macs. ^∗∗^p < 0.01, ^∗∗∗∗^p < 0.0001, Student’s t test compared with SD control. Error bars indicate ±SEM. (D) Tile scan of liver from a WD-fed mouse (36 weeks; same mouse as from A and B) with identified Desmin^hi^ regions demarcated in blue. The rest of the tissue is classified as Desmin^lo^, excluding the larger vessels, for quantification in (E). (E) Quantification of indicated populations in Desmin^hi^ and Desmin^lo^ zones (from D) as a % of each mac subset. ^∗^p < 0.05, Student’s t test compared with Desmin^lo^ area. Quantification data in (C) and (E) are pooled from 2 independent experiments with n = 6. Error bars indicate ±SEM. (F–I) Monocyte- and mac-containing clusters (based on expression of *Mafb*, *Ly6c2*, *Ccr2*, *Fcgr1*, *Adgre1*) were isolated from the CITE-Seq data (18,241 cells) and re-clustered. (F) UMAP showing annotated monocyte and macrophage clusters. (G) Distribution of cells on SD or WD at indicated time points, with SD data coming from cells pooled after 12, 24, and 36 weeks. (H and I) Expression of indicated genes by the different clusters (SD + WD pooled). See also Figure S5.

Having identified changes in the ResKC niche in MAFLD, we next set out to explore whether resident and recruited macs occupied the same or distinct niches in MAFLD. We therefore localized CLEC4F^+^TIM4^+^ ResKCs, CLEC4F^+^TIM4^-^ moKCs, and CLEC4F^-^ macs using confocal microscopy after feeding the SD or WD for 12, 24, and 36 weeks. This revealed that all MAFLD macs, irrespective of CLEC4F and TIM4 expression, were located in the zones between the portal and central veins, with at least a part of their body in contact with LSECs (CD31^+^) and hepatic stellate cells (Desmin^+^) (Figures 4A–4C). This location was identical to that observed for ResKCs in healthy liver (Bonnardel et al., 2019). Notably, there were also some CLEC4F^-^ macs found in close proximity to the large vessels (central & portal veins), but these were also found in the SD-fed mice (Figure 4B). Automated identification of the macs based on expression of F4/80 followed by classification into TIM4^+^ KCs, TIM4^-^ KCs, or CLEC4F^-^ macs identified similar proportions of the different subsets as retrieved by flow cytometry (Figures 4B, 4C, and S3B). However, this identified regions where CLEC4F^-^ macs were the main mac subset present (Figures 4A and 4B). These zones were characterized by increased Desmin expression (Figures 3D and 3E), a characteristic of fibrotic stellate cells and myofibroblasts (Ballardini et al., 1988). Thus, these findings suggest that recruited macs populate the ResKC niche in MAFLD, with CLEC4F^-^ macs being predominantly found in fibrotic zones.

### Recruited Macs Exist in Distinct Subsets

We next investigated the similarities and differences between CLEC4F^-^ macs and KCs. To this end, we isolated cells of the monocyte and mac lineages from the CITE-seq data (18,241 cells) and re-clustered them (Figures S5A and 4F, www.livercellatlas.org). The sub-clusters revealed by this additional analysis varied between diets and time points (Figures 4G and S5B). Analysis of the DEGs and surface proteins expressed per cluster allowed them to be identified more precisely (Figures 4H, 4I and S5C–S5E). Clusters 0, 1, 3, 5, 14, and 17 were monocyte-like cells (Figures 4F, S5D, and S5E; Table S3), and of these, cluster 3 was the main population in the SD-fed mice, whereas cluster 17 was a minor population, present in all conditions but increased in WD-fed mice (Figures 4G and S5B). Cells in cluster 17 were enriched for IFN-induced genes, including *Ifit1*, *Ifit3*, and *Isg15* (Figure S5D; Table S3), similar to a subset of monocytes recently reported (Ydens et al., 2020). Cluster 11 was identified as patrolling Ly6C^lo^ monocytes, while clusters 4, 7, 10, and 12 had features of monocytes and macs, suggesting that they were transitioning monocytes differentiating into macs (Figures 4F, 4I, S5D, and S5E; Table S3). Clusters 6, 8, 9, 15, 22, and 23 were identified as ResKCs on the basis of their expression of *Clec4f* and ResKC genes (Beattie et al., 2016; Scott et al., 2016), including *Timd4*, *C2, Xlr, Marco* & *Cd163* (Figures 4H, S5D, and S5E; Table S3). Of these, cluster 6, enriched for genes associated with inflammation, was restricted to the mouse fed the SD for 36 weeks (Figures 4G, S5B, and S5F; Table S4). However, no significant differences in the expression of these genes were observed by qPCR (Figure S5G).

Clusters 2, 13, 16, 18, 19, 20, and 21 were also identified as macs. Of these, clusters 19 and 21 were proliferating cells containing *Clec4f*^+^ KCs and *Clec4f*^-^ macs (Figures 4H, S5D, and S5E). Cells in cluster 13 were identified as moKCs, expressing *Clec4f* and many other KC signature genes, but lacking expression of ResKC genes such as *Timd4* (Figures 4H, S5D, and S5E). Based on a list of capsule mac signature genes (Table S4) generated by comparing the gene expression profile of liver capsule macs (Sierro et al., 2017) with those of ResKCs and Ly6C^hi^ monocytes (Scott et al., 2016) and mapping the signature onto our data using the signature finder algorithm (Pont et al., 2019), cluster 20 was identified as liver capsule macs (Figure S5C). Clusters 2 and 16, which were specific to WD-fed mice, could not yet be further identified and were called Mac1 and Mac2, respectively (Figure 4F). Cluster 18 identified a group of cells that shared expression of *Mmp12* (Figures S5D and S5E). However, mapping individual cells in this cluster onto the other clusters using the FindTransferAnchors function of the Seurat R package showed that this was a heterogeneous population of ResKCs, moKCs, Mac1s, and Mac2s; thus, for further analysis, they were included with the cells of the same identity.

To determine which clusters represented the macs identified in our flow cytometry analysis, we purified the 3 subsets based on CLEC4F and TIM4 from SD- and WD-fed mice (12 and 24 weeks) and performed bulk RNA-seq (Figure S6A). PCA analysis and comparison of the 3 populations in a triwise plot revealed that the majority of DEGs between the 3 populations were either specific to the CLEC4F^-^ macs (*Spp1, Chil3, Ccr2,* and *Gpr183*) or shared between the moKCs and ResKCs with the exception of residency genes (Beattie et al., 2016; Scott et al., 2016) specific to the ResKCs, including *Timd4, Cd163, C6, Xlr*, and *Marco* (Figure S6A). To define the signature of each population, we compared the cells to one another and took the genes with a Log fold change (FC) >2. This process was refined for the CLEC4F^-^ macs by an additional comparison of their DEGs with BM monocytes. Similarly, an additional comparison was carried out between moKCs and ResKCs, to ensure selection of DEGs that were unique to the moKC population. Using the signature finder algorithm (Pont et al., 2019) to map the resulting signatures (Table S5) onto the scRNA-seq data (Figure S6B), we confirmed the annotations of the populations in the scRNA-seq data. Notably, while the CLEC4F^-^ macs mapped primarily onto the Mac2s, they also showed some similarities to the Mac1s (Figure S6B), indicating that the CLEC4F^-^ cells are heterogeneous.

### Hepatic Lipid-Associated Macs Differentiate in MAFLD

**Figure 5:**
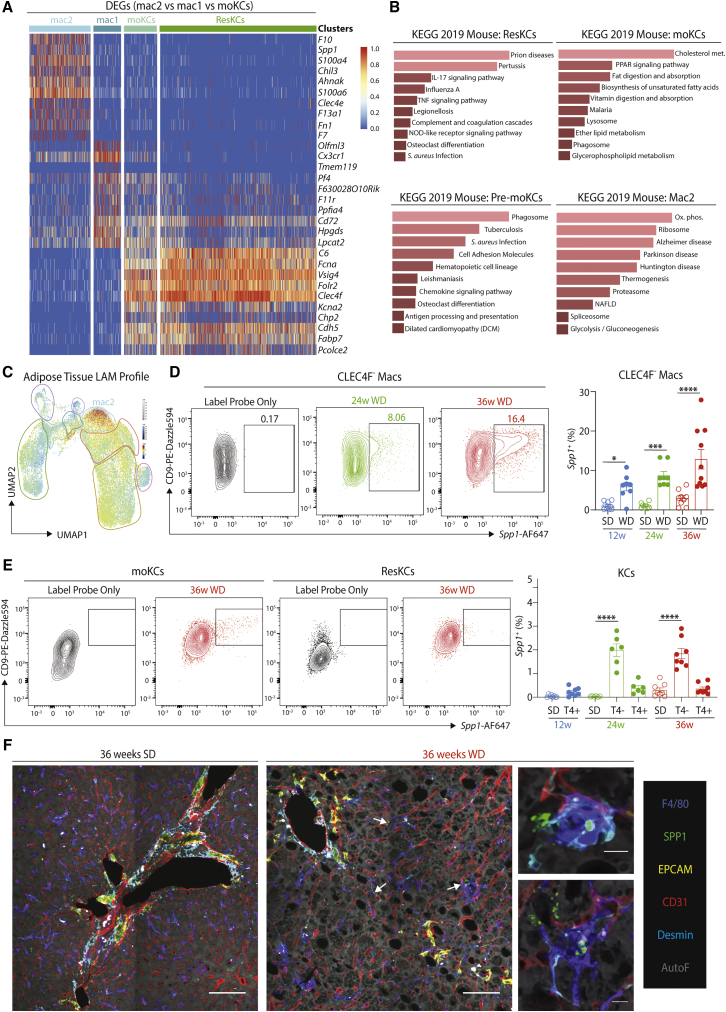
Hepatic LAMs in MAFLD Are Identified by Spp1 Expression (A) Heatmap showing DEGs between Mac2, Mac1, and moKC populations from mice fed the WD for 24 and 36 weeks pooled and their expression by indicated populations. (B) KEGG pathway analysis on DEGs for each indicated subset (see Tables S1 and S3). (C) The adipose tissue LAM signature (Jaitin et al., 2019) was mapped onto the liver mac UMAP to identify cells with a similar profile using the Signature Finder algorithm (Pont et al., 2019). (D and E) Expression of *Spp1* by CLEC4F^-^ macrophages (D) and moKCs and ResKCs (E) at 24 and 36 weeks on WD as measured by Prime Flow (left panels, representative plots), and right, proportions of indicated populations (T4 = TIM4). Data are pooled from 2 experiments with 6 mice per group. ^∗^p < 0.05, ^∗∗∗^p < 0.001, ^∗∗∗∗^p < 0.0001. One-way ANOVA. Error bars indicate ±SEM. (F) Confocal microscopy (2x2 Tiles) showing expression of F4/80 (blue), SPP1 (green), EPCAM (yellow), CD31 (red), Desmin (cyan), and tissue autofluorescence (gray) in livers of SD- and WD-fed mice (36 weeks). Scale bar, 100 μm. White arrows identify SPP1^+^ macrophages. Inset shows zoomed in images showing colocalization of SPP1 and F4/80 signal (36 weeks WD). Scale bar, 10 μm. Images are representative of 6 mice from 2 independent experiments. See also Figure S6.

To investigate the heterogeneity of the CLEC4F^-^ macs, we used triwise plots to define the DEGs that separated the as yet unidentified Mac1s and Mac2s from the other recruited population, the moKCs (Figure S6C). Comparison of these DEGs with ResKCs confirmed the similarity between moKCs and ResKCs, while the 2 unidentified mac clusters were distinct (Figure 5A; Table S4). The Mac1s were enriched for genes such as *Cx3cr1, Itgax, H2-M2*, and *Olfml3*, while the Mac2s were enriched for *Spp1, F10, Chil3, Fabp5*, and *Gpnmb* expression (Figures 5A and S6C–S6E). Notably, the gene expression profile of Mac1s somewhat resembles moKCs (Figure 5A) and macs *en route* to becoming moKCs (Bonnardel et al., 2019). While this manuscript was under review, *Clec1b* (encoding CLEC2) was proposed to be a good marker of moKCs that appeared to be expressed earlier than CLEC4F (Tran et al., 2020). To confirm this, we examined expression of CLEC4F and CLEC2 in *Clec4f*-DTR mice (Scott et al., 2016) 3 and 6 days post-ResKC depletion during the generation of moKCs (Scott et al., 2016). This analysis confirmed CLEC2 to be an early marker of macs becoming moKCs, and hence, we termed the CLEC2^+^CLEC4F^-^ macs “pre-moKCs” (Figure S6F). As the Mac1s seemed to resemble macs *en route* to becoming moKCs, we checked if these would be pre-moKCs. Fitting with the scRNA-seq data, the CLEC4F^-^ macs could be split into 2 populations based on CLEC2 expression (Figures S6D and S6G). Thus, Mac1s are pre-moKCs. To define Mac2s, we next performed KEGG pathway analysis (Table S4). This suggested the Mac2s to be relevant in diseases, including MAFLD (Figure 5B). While *Cd9* and *Trem2* expression was increased across mac subsets in MAFLD (Table S6), the overall signature of the Mac2s closely resembled those of *Cd9-* and *Trem2*-expressing lipid-associated macrophages (LAMs) described recently in obese adipose tissue (AT; Figure S6H) (Hill et al., 2018; Jaitin et al., 2019), suggesting that this cluster may be their hepatic counterpart. Indeed, when we calculated the signature of the AT LAMs compared with other AT macs (Jaitin et al., 2019) (Table S7), the signature finder algorithm (Pont et al., 2019) mapped the LAM signature onto the Mac2s (Figure 5C). Thus, we called these cells hepatic LAMs. Comparison of the DEGs between hepatic and AT LAMs identified 67 DEGs with a Log FC >1.5, indicating tissue-specific influences on the differentiation of these cells (Figure S6I; Table S7). The hepatic LAMs also showed some overlap with the scar-associated macs that have recently been described in fibrotic human livers (Ramachandran et al., 2019) and whose signature is also enriched for expression of *TREM2* and *CD9* (Figure S6J). Examination of the CITE-seq data did not reveal any specific antibodies for phenotypic analysis of the hepatic LAMs, while flow cytometry analysis of potential surface markers showed reactivity that was either non-selective (e.g. CD9) (Figures 5D and 5E) or gave no signal above controls (GPNMB, CD63; data not shown). Thus, we next examined other suitable markers of the LAM population and identified *Spp1* to be specifically enriched in LAMs (Figure 4I). As *Spp1* encodes the chemokine osteopontin, implicated in fibrosis and NASH (Arriazu et al., 2017; Glass et al., 2018), we tested whether it could be used as a marker of hepatic LAMs. Using the PrimeFlow assay to measure mRNA transcripts by flow cytometry, we detected *Spp1* primarily in CLEC4F^-^ macs, identifying the hepatic LAMs (Figures 5D and 5E). While the SPP1 antibody proved unsuitable for flow cytometry, we were able to confirm the presence of SPP1-expressing macs in MAFLD by immunofluorescence, using EPCAM to exclude SPP1-expressing cholangiocytes (Figures 5F and 3B).

### Hepatic LAMs and KCs Have Distinct Abilities to Metabolize Lipid

**Figure 6:**
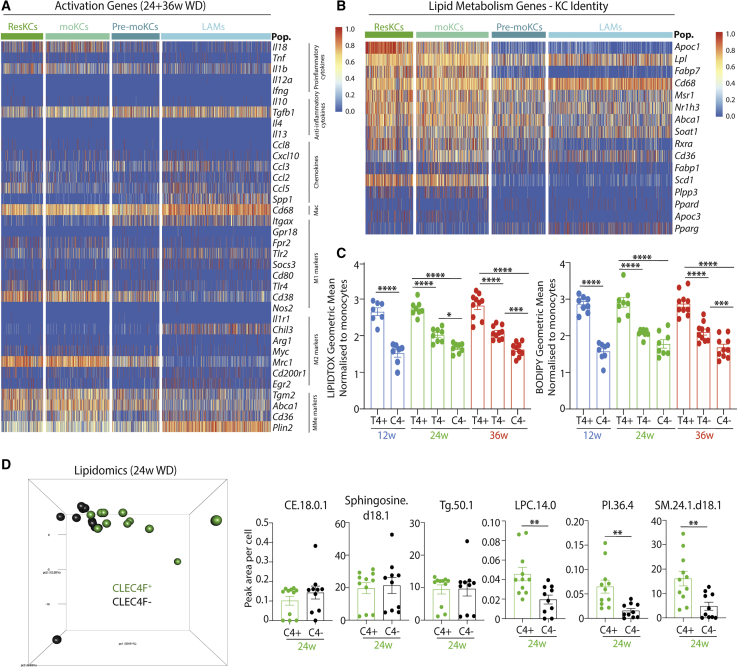
Characterization of Recruited Macrophages in MAFLD (A) Heatmap showing expression of immune activation-associated genes in ResKCs, moKCs, pre-moKCs and hepatic LAMs from WD-fed mice (24 and 36 weeks, pooled). (B) Heatmap showing expression of genes associated with lipid metabolism previously reported to be enriched in ResKCs (Scott et al., 2016) in ResKCs, moKCs, pre-moKCs, and hepatic LAMs from WD-fed mice (24 and 36 weeks, pooled). (C) Neutral lipid content of ResKCs (T4+), moKCs (T4-), and CLEC4F^-^ macs (C4-) after 12, 24, and 36 weeks on WD. Results shown are geometric mean for Lipidtox and BODIPY staining normalized to Ly6C^hi^ monocytes from the same liver. ^∗^p < 005, ^∗∗∗^p < 0.001, ^∗∗∗∗^p < 0.0001. One-way ANOVA compared with ResKCs at each time point. Error bars indicate SEM. (D) Lipidomics analysis of sorted CLEC4F^+^ KCs and CLEC4F^-^ macs from WD-fed mice (24 weeks). Left: PCA plot showing results for different macs. Right: indicated lipid species in CLEC4F^+^ KCs (C4+) or CLEC4F^-^ macs (C4-). ^∗∗^p < 0.01 Student’s t test. Error bars indicate ±SEM. See also Figure S6.

We next sought to investigate how the different macs compared functionally. We first examined expression of a list of generic prototypical immune activation-associated genes in hepatic LAMs, pre-moKCs, moKCs, and ResKCs pooled from mice fed the WD (24 and 36 weeks). Overall, the different KCs expressed these genes similarly, while hepatic LAMs expressed lower *Il18, Fpr2, Tlr4, Cd38, Tgm2, Mrc1*, and *Abca1* and higher *Spp1, Ccl3, Itgax, Socs3, Chil3*, and *Plin2* (Figures 6A and S7A). We next examined expression of lipid metabolism genes, as this is proposed to be a main function of ResKCs. This revealed a similar pattern in which moKCs were similar to ResKCs, with the exception of *Apoc1* (lower) and *Pparg* (higher), whereas LAMs showed lower or equal expression of most of these genes compared with the KC populations, with the exception of *Lpl* & *Pparg* (Figures 6B and S7B). Pre-moKCs had an intermediate phenotype between the KCs and the LAMs (Figure 6B). Consistent with these findings, CLEC4F^-^ macs contained fewer neutral lipids than their ResKC or moKC counterparts (Figure 6C), as measured by BODIPY or LIPIDTOX staining. Despite containing more neutral lipid than CLEC4F^-^ macs, moKCs also contained less lipid than the ResKCs (Figure 6C). To examine this further, we performed lipidomic analysis. Due to a number of factors including cell numbers required, the lack of a *Spp1* antibody, the lack of knowledge of CLEC2 expression at the time of analysis, and the overall similarity between ResKCs and moKCs, we compared total CLEC4F^+^ KCs with CLEC4F^-^ macs. This showed that although KCs and CLEC4F^-^ macs shared some lipid species, others were found only in KCs and not in CLEC4F^-^ macs (Figure 6D), further suggesting that these cells have distinct abilities to metabolize lipids. Thus, hepatic LAMs and moKCs represent distinct fates for monocytes recruited to the liver in MAFLD in terms of their transcriptome, localization, and function.

### ResKCs Are Not Pro-inflammatory in MAFLD

**Figure 7:**
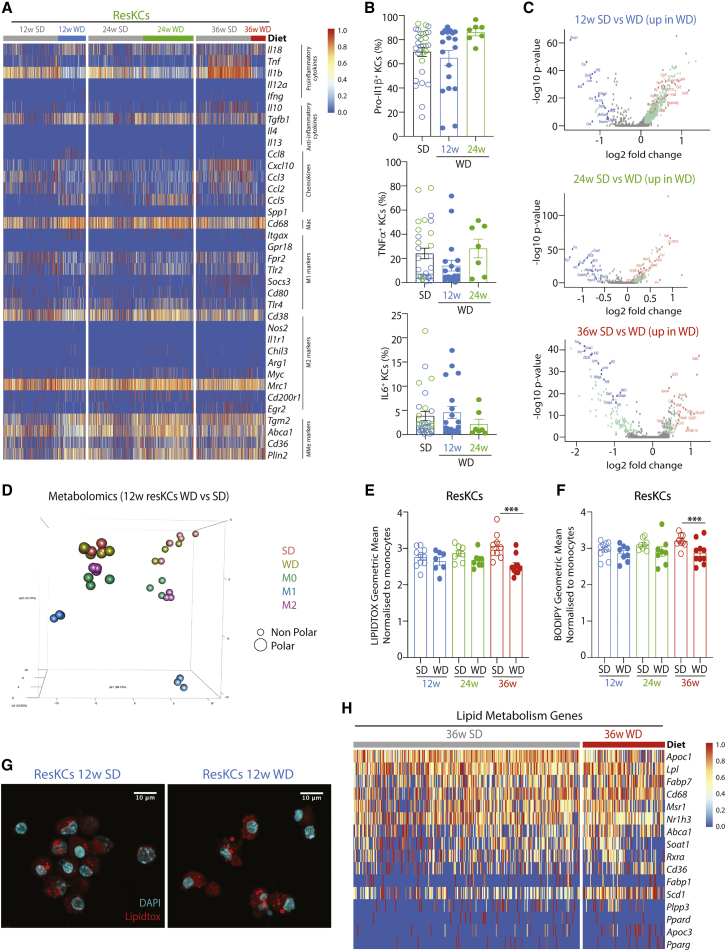
Features of ResKCs in MAFLD (A) Heatmap showing expression of immune activation-associated genes by ResKCs after 12, 24, and 36 weeks on the WD, compared with SD (gray) from the same time point. (B) Pro-inflammatory cytokine expression by ResKCs at indicated time points in either SD- or WD-fed mice as measured by intracellular cytokine staining. Data are pooled from 2 experiments, with n = 7–19 mice per group. Error bars indicate ±SEM. (C) Volcano plots showing DEGs between ResKCs (SD or WD) for indicated time points. (D) PCA plot showing metabolomic analysis (non-polar and polar metabolites) of ResKCs after 12 weeks on SD or WD compared with *in vitro* polarized M0, M1 or M2 BM-derived macrophages. Data are from a single experiment, with n = 3–5 per group. (E and F) Neutral lipid content of ResKCs after 12, 24, and 36 weeks on WD compared with SD. Results shown are geometric mean for (E) Lipidtox and (F) BODIPY staining normalized to Ly6C^hi^ monocytes from the same livers. ^∗^p < 0.05. One-way ANOVA compared with SD at each time point. Error bars indicate ±SEM. (G) Sorted ResKCs from SD- or WD-fed mice (12 weeks) were allowed to adhere to a coverslip and stained for Lipidtox and DAPI and imaged by confocal microscopy. (H) Heatmap showing expression of genes associated with lipid metabolism profile in ResKCs from the mice fed either the SD or WD (36 weeks). See also Figure S7.

Finally, we examined how ResKCs responded in MAFLD. The gradual loss of ResKCs during MAFLD indicates that these cells are affected by the lipemic environment, and it is generally believed that immune activation of KCs occurs in MAFLD (Krenkel and Tacke, 2017). However, the exact effects of MAFLD on KCs are contentious (Morgantini et al., 2019; Xiong et al., 2019). Consistent with a recent report (Morgantini et al., 2019), we did not observe immune activation of KCs at any stage of MAFLD in either the scRNA-seq or bulk RNA-seq analysis, as evidenced by expression of genes associated with immune cell activation. Although we observed a slight decrease in *Tnfa, Il1b*, and *Il10* expression in ResKCs from WD-fed mice (Figures 7A and S7C), no differences were found in pro-inflammatory cytokine production (Figure 7B), thus demonstrating that ResKCs are not pro-inflammatory in MAFLD. Furthermore, although changes could be seen in the ResKC transcriptome when examining all the DEGs between SD- and WD-fed mice at the different time points, the scale of these changes was modest compared with the differences between KCs and CLEC4F^-^ macs (Table S6). Using a log FC >1.5 as a cut-off, analysis of the scRNA-seq data revealed 2 DEGs in ResKCs at 12 weeks, 0 DEGs at 24 weeks, and 13 DEGs at 36 weeks (Figure 7C). Analysis of the more in-depth bulk RNA-seq data showed 1 DEG in WD ResKCs at 12 weeks and 100 DEGs at 24 weeks, compared with 781 DEGs between WD ResKCs and CLEC4F^-^ macs at 24 weeks (Figure S7D; Table S6). KEGG pathway analysis suggested that many of the upregulated genes in WD ResKCs were associated with cell adhesion and cytokine-receptor interactions (data not shown). There was also some increased expression of genes associated with metabolically activated macs (MMe) in ResKCs from mice with MAFLD (Figures 7A and S7C); however, this did not correlate with a difference in the ResKC metabolome between SD and WD (12 weeks), although the ResKC profile was distinct from *in vitro* M0, M1, and M2 macrophages (Figures 7D and S7F). Moreover, LIPIDTOX and BODIPY staining showed that the neutral lipid content of ResKCs did not increase in WD-fed mice at 12, 24, or 36 weeks (Figures 7E–7G); rather, there was a slight decrease after 36 weeks (Figures 7E and 7F). Analysis of the ResKC lipid metabolism profile at the different time points on the diet identified a trend toward slightly increased expression of these genes in WD-fed mice (Figures 7H and S7E), possibly compensating for the increased lipid load. Focused ion beam milling combined with scanning electron microscopy (FIB-SEM) and lipidomics analysis also did not identify any overt differences in either lipid droplet volume or in the lipid species present in CLEC4F^+^ KCs isolated from mice fed the SD or WD (Figure S7G; data not shown). Taken together, this suggests that while ResKCs react in MAFLD, they are not pro-inflammatory, nor do they harbor an increased or altered lipid load. Thus, the biggest difference in MAFLD macs stems from the recruitment of LAMs which are distinct from ResKCs, moKCs, and pre-moKCs, highlighting the need to differentiate between these mac subsets.

## Discussion

MAFLD represents a spectrum of disease states ranging from simple steatosis to NASH, which can lead to cirrhosis and HCC. However, not all patients progress from MAFLD to NASH, and while the reasons why some do and some don’t remain largely unclear, hepatic macs have been proposed to play a role in this. Recently, it has become clear that hepatic macs, especially in disease settings, do not represent a homogeneous population of ResKCs but rather can exist in multiple subsets and/or activation states (Remmerie et al., 2020; Xiong et al., 2019). To begin to assess the roles played by the distinct mac populations in MAFLD, we set out to characterize mac heterogeneity and localize the subsets within the liver. We identified four subsets of hepatic macs in MAFLD, including ResKCs and 3 subsets of recruited macs, moKCs, pre-moKCs, and the distinct hepatic LAMs. Notably, all populations were found within the KC niche in contact with hepatic stellate cells (HSCs) and liver sinusoidal endothelial cells (LSECs) (Bonnardel et al., 2019). However, LAMs were predominantly found in regions characterized by increased Desmin expression, suggestive of fibrosis. During reviewing, two additional papers were published investigating macs in MAFLD (Seidman et al., 2020; Tran et al., 2020). It is currently unclear exactly how the populations identified here correlate to those described by Seidman et al. (2020). Comparison of the datasets would suggest that the hepatic LAMs would most likely be present in the KN-RM gate; however, with the expression pattern of F4/80, TIM4, and CD11b, some LAMs may also be in the Ly6C^lo^-RM gate (Seidman et al., 2020). However, as distinct diets were used, this may also affect the populations present. With this in mind, it will also be important to examine whether LAMs are found within CLEC2^-^ macs recruited in the MCD-model (Tran et al., 2020). Additionally, Xiong et al. (2019) have also described a population of macs termed NASH-associated macs (NAMs). In contrast to our study, the NAMs were identified as KCs. However, only a subset of these cells expressed KC identity genes like *Cd5l* or *Clec4f* (Remmerie et al., 2020; Xiong et al., 2019), suggesting that these may also include LAMs.

How important is the distinction between the recruited mac subsets? While others have suggested minimal differences between TIM4^-^ macs and ResKCs in NASH (Seidman et al., 2020), by dividing the population into moKCs and LAMs, we identified that moKCs largely resembled ResKCs, while LAMs had more than 700 DEGs compared with ResKCs, including differences in lipid metabolism and immune activation. Moreover, as the hepatic LAMs best resemble scar-associated macrophages described in fibrotic human livers (Ramachandran et al., 2019), this distinction is also clinically relevant. Moreover, hepatic LAMs expressed *Spp1* encoding the chemokine Osteopontin. Osteopontin has recently been described as a good biomarker of NASH in patient serum (Glass et al., 2018). Thus, as hepatic LAMs were only identified in the later stages of disease correlating with worsening disease and fibrosis, it is tempting to speculate that this increase in osteopontin observed in patients could be attributed to the hepatic LAMs. In addition, osteopontin has been implicated in driving collagen I production from HSCs (Seth et al., 2014) and thus is thought to contribute to fibrosis (Arriazu et al., 2017). This could suggest that hepatic LAMs drive the progression to NASH. Indeed, recent studies blocking osteopontin in mice models of NASH have suggested a protective effect (Coombes et al., 2016; Honda et al., 2020; Kiefer et al., 2010). Unfortunately, to date, we do not have the tools to target this population specifically to address these questions, but generating these is an important aim for the future.

Turning our attention to the ResKCs, we found that, contrary to the current line of thinking (Reid et al., 2016; Tosello-Trampont et al., 2012; Krenkel and Tacke, 2017), ResKCs are not pro-inflammatory in MAFLD. Thus, this questions the proposed role of KCs as drivers of NASH, through their role as inflammatory mediators (Krenkel and Tacke, 2017). While not pro-inflammatory, ResKCs had an altered transcriptome in MAFLD; however, the scale of these changes was modest compared with differences between ResKCs and LAMs. Moreover, minor, if any, changes were observed in the metabolomics and lipidomics profiles of KCs. One possibility is that the modest response observed here is because we can only profile the remaining ResKCs. Perhaps if we were to catch the ResKCs just prior to their loss, increased differences would be observed. The modest response of ResKCs is in contrast to a recent report, where over 800 DEGs genes have been identified between NASH and healthy ResKCs. For example, we did not observe the altered expression of *C6 o*r *Cd163* (Seidman et al., 2020). The reasons for these differences remain unclear but could be related to the timing or diet used. As many KC residence and identity genes were downregulated in NASH TIM4^+^ KCs in the study from Seidman et al. (2020), one possibility is that the proportion of moKCs acquiring TIM4 expression may be different between the models, leading to a more immature KC profile in NASH in the study from the Glass lab. Indeed, many of the DEGs reported between NASH and healthy KCs, including *C4b, Timd4, Marco*, and *Hmox1*, were dysregulated between our moKCs and ResKCs. However, overall, the differences between moKCs and ResKCs in our model were also relatively modest (72 DEGs, LogFC > 1.5).

Despite these differences, our observation that ResKCs were gradually lost from the tissue in MAFLD is consistent with the other recent studies (Seidman et al., 2020; Tran et al., 2020). Regarding why ResKCs fail to self-maintain in MAFLD, one hypothesis is that they are no longer correctly adapted to the niche. While our data did not identify any major changes in the niche signals important for KC development and maintenance (Bonnardel et al., 2019; Sakai et al., 2019), we observed an increase in niche cell proliferation, suggesting an increased need to maintain cell numbers in MAFLD. Moreover, we also identified differences in the transcriptomes of these cells. However, further work is required to better understand the precise signals at play. With the knowledge that ResKCs have been suggested to be protective in NASH (Tran et al., 2020), our data suggesting a lack of ResKCs in HCC and the finding that osteopontin expression correlates with worse disease (Glass et al., 2018), perhaps if we can understand the signals driving ResKC loss and LAM generation in MAFLD, this could open the door to therapeutic options for patients to prevent and/or reverse progression to NASH and HCC.

Together, our data highlight the heterogeneity within the macrophage pool in MAFLD and highlight the need for examination of this heterogeneity when considering therapeutic options.

## STAR★Methods

### Key Resources Table

**Table:** 

REAGENT or RESOURCE	SOURCE	IDENTIFIER
**Antibodies**		

Armenian Hamster Monoclonal CD103-PE (clone 2E7)	eBioscience	12-1031-82; RRID: AB_465799
Rat Monoclonal CD11b - BV605 (clone M1/70)	BD Horizon	563015; RRID: AB_2737951
Rat Monoclonal CD11b - PE-Cy7 (clone M1/70)	BD PharMingen	552850; RRID: AB_394491
Armenian Hamster Monoclonal CD11c - PE-eFluor610 (clone N418)	eBioscience	61-0114-82; RRID: AB_2574530
Rat Monoclonal CD172a - BB630P (clone P84)	BD Customs	624294
Rat Monoclonal CD19 - PE-Cy5 (clone 1D3)	eBioscience	15-0193-82 ; RRID: AB_657672
Rat Monoclonal CD206 - APC (clone C068C2)	BioLegend	141708; RRID: AB_10900231
Rat Monoclonal CD26 - FITC (clone H194-112)	BD PharMingen	559652; RRID: AB_397295
Rat Monoclonal CD31 - Unconjugated (clone MEC 13.3)	BD PharMingen	550274; RRID: AB_393571
Rat Monoclonal CD38 - AF700 (clone 90)	eBioscience	56-0381-82; RRID: AB_657740
Armenian Hamster Monoclonal CD3e - PE-Cy5 (clone 145-2C11)	TONBOBiosciences	55-0031; RRID: AB_2621815
Rat Monoclonal CD43 - BUV737 (clone S7)	BD Horizon	612840; RRID: AB_2738790
Rat Monoclonal CD45 - BV510 (clone 30-F11)	BioLegend	103138; RRID: AB_2563061
Rat Monoclonal CD45 - PE-Cy7 (clone 30-F11)	eBioscience	25-0451-82; RRID: AB_469625
Mouse Monoclonal CD45.1 - PE (clone A20)	BD PharMingen	553776; RRID: AB_395044
Mouse Monoclonal CD45.2 - AF700 (clone 104)	eBioscience	56-0454-82; RRID: AB_657752
Rat Monoclonal CD45R - PE-Cy5 (clone RA3-6B2)	BD Biosciences	553091; RRID: AB_394621
Mouse Monoclonal CD64 - AF647 (clone X54-5/7.1)	BD PharMingen	558539; RRID: AB_647120
Mouse Monoclonal CD64 - BV711 (clone X54-5/7.1)	BioLegend	139311; RRID: AB_2563846
Rat Monoclonal CD9 - PE (clone MZ3)	BioLegend	124805; RRID: AB_1279327
Rat Monoclonal CD9 - PE/Dazzle594 (clone MZ3)	BioLegend	124822; RRID: AB_2800602
Rat Monoclonal CLEC2 - PE (clone 17D9)	Biolegend	146104; RRID: AB_2562382
Goat Polyclonal Clec4F - Unconjugated	R & D Systems	AF2784; RRID: AB_2081339
Rat Monoclonal Clec4F - Unconjugated (clone 370901)	R & D Systems	MAB2784; RRID: AB_2081338
Rabbit Polyclonal Desmin - Unconjugated	Abcam	ab15200; RRID: AB_301744
Donkey Anti-Goat IgG - AF488	Invitrogen	A-11055; RRID: AB_2534102
Donkey Anti-Goat IgG - AF555	Invitrogen	A-21432; RRID: AB_2535853
Donkey Anti-Goat IgG - AF647	Invitrogen	A-21447; RRID: AB_2535864
Donkey Anti-Rat IgG - Cy3	Jackson ImmunoResearch	712-166-153; RRID: AB_2340669
Rat Monoclonal Epcam - APC (clone G8.8)	eBioscience	17-5791-82; RRID: AB_2716944
Rat Monoclonal F4/80 - Biotin (clone BM8)	eBioscience	13-4801-85; RRID: AB_466657
Rat Monoclonal F4/80 - BV785 (clone BM8)	BioLegend	123141; RRID: AB_2563667
Rat Monoclonal F4/80 - eFluor450 (clone BM8)	eBioscience	48-4801-82; RRID: AB_1548747
Rabbit Polyclonal Glutamine Synthetase - Unconjugated	Abcam	ab73593: RRID: AB_2247588
Goat Anti-Rabbit IgG - AF514	Invitrogen	A-31558; RRID: AB_2536173
Rat Monoclonal IL12/IL23p40 - eFluor450 (clone C17.8)	eBioscience	48-7123-82; RRID: AB_2574111
Rat Monoclonal IL1b - APC-eFluor780 (clone NJTEN3)	eBioscience	47-7114-82; RRID: AB_2573996
Rat Monoclonal IL-6 - PE (clone MP5-20F3)	eBioscience	12-7061-82; RRID: AB_466165
Mouse Monoclonal Ki67 antigen - BV786 (clone B56)	BD Horizon	563756; RRID: AB_2732007
Rat Monoclonal Ki67 antigen - Unconjugated (clone TEC-3)	Agilent Dako	M7249; RRID: AB_2250503
Rat Monoclonal Ly6C - Biotin (clone AL–21)	BD PharMingen	557359; RRID: AB_396663
Rat Monoclonal Ly6C - eFluor450 (HK1.4)	eBioscience	48-5932-82; RRID: AB_10805519
Rat Monoclonal Ly6G - AF700 (clone 1A8)	BD PharMingen	561236; RRID: AB_10611860
Rat Monoclonal Ly6G - BUV395 (clone 1A8)	BD Horizon	563978: RRID: AB_2716852
Rat Monoclonal Ly6G - BUV563 (clone 1A8)	BD Horizon	612921: RRID: AB_2739334
Ra Monoclonal Lyve-1 - Biotin (ALY7)	eBioscience	13-0443-82; RRID: AB_1724157
Rat Monoclonal MGL1 - AF647 (LOM-8.7)	BioLegend	145603; RRID: AB_2561986
Rat Monoclonal MHCII - AF700 (clone M5/114.15.2)	eBioscience	56-5321-82; RRID: AB_494009
Rat Monoclonal MHCII - APC-eFluor780 (clone M5/114.15.2)	eBioscience	47-5321-82; RRID: AB_1548783
Rat Monoclonal MHCII - BUV805 (clone M5/114.15.2)	BD Customs	624287
Mouse Monoclonal NK1.1 - PE-Cy5 (clone PK136)	BioLegend	108716; RRID: AB_493590
Rat Monoclonal SiglecF - BUV395 (clone E50-2440)	BD Biosciences	740280; RRID: AB_2740019
Goat Polyclonal Spp1 - Unconjugated	R & D Systems	AF808; RRID: AB_2194992
Mouse Monoclonal T-bet - PE-Cy7 (clone 4B10)	eBioscience	25-5825-82; RRID: AB_11042699
Rat Monoclonal Ter119 - PE-Cy5 (clone TER-119)	eBioscience	15-5921-82; RRID: AB_468810
Rat Monoclonal Tim4 - AF647 (clone RMT4-54)	BioLegend	130008; RRID: AB_2271648
Rat Monoclonal Tim4 - PerCP-Cy5.5 (clone RMT4-54)	eBioscience	46-5866-82; RRID: AB_2573781
Rat Monoclonal TNFa - APC (MP6-XT22)	BD PharMingen	554420; RRID: AB_398553
Mouse Monoclonal XCR1 - BV650 (ZET)	BioLegend	148220; RRID: AB_2566410
TotalSeq™-A0001 anti-mouse CD4 Antibody (clone RM4-5)	BioLegend	100569; RRID: AB_2749956
TotalSeq™-A0002 anti-mouse CD8a Antibody (clone 53-6.7)	BioLegend	100773; RRID: AB_2734151
TotalSeq™-A0003 anti-mouse CD366 (Tim-3) Antibody (clone RMT3-23)	BioLegend	119729; RRID: AB_2734178
TotalSeq™-A0004 anti-mouse CD279 (PD-1) Antibody (clone RMP1-30)	BioLegend	109123; RRID: AB_2734169
TotalSeq™-A0012 anti-mouse CD117 (c-kit) Antibody (clone 2B8)	BioLegend	105843; RRID: AB_2749960
TotalSeq™-A0013 anti-mouse Ly-6C Antibody (clone HK1.4)	BioLegend	128047; RRID: AB_2749961
TotalSeq™-A0014 anti-mouse/human CD11b Antibody (clone M1/70)	BioLegend	101265; RRID: AB_2734152
TotalSeq™-A0015 anti-mouse Ly-6G Antibody (clone 1A8)	BioLegend	127655; RRID: AB_2749962
TotalSeq™-A0070 anti-human/mouse CD49f Antibody (clone GoH3)	BioLegend	313633; RRID: AB_2734291
TotalSeq™-A0073 anti-mouse/human CD44 Antibody (clone IM7)	BioLegend	103045; RRID: AB_2734154
TotalSeq™-A0074 anti-mouse CD54 Antibody (clone YN1/1.7.4)	BioLegend	116127; RRID: AB_2734177
TotalSeq™-A0076 anti-mouse/human CD15 (SSEA-1) Antibody (clone MC-480)	BioLegend	125615; RRID: AB_2800603
TotalSeq™-A0077 anti-mouse CD73 Antibody (clone TY/11.8)	BioLegend	127227; RRID: AB_2749980
TotalSeq™-A0078 anti-mouse CD49d Antibody (clone R1-2)	BioLegend	103623; RRID: AB_2734159
TotalSeq™-A0079 anti-mouse CD200 (OX2) Antibody (clone OX-90)	BioLegend	123811; RRID: AB_2734191
TotalSeq™-A0090 Mouse IgG1, κ isotype Ctrl Antibody (clone MOPC-21)	BioLegend	400199
TotalSeq™-A0091 Mouse IgG2a, κ isotype Ctrl Antibody (clone MOPC-173)	BioLegend	400285
TotalSeq™-A0092 Mouse IgG2b, κ isotype Ctrl Antibody (clone MPC-11)	BioLegend	400373
TotalSeq™-A0093 anti-mouse CD19 Antibody (clone B4)	BioLegend	115559; RRID: AB_2749981
TotalSeq™-A0094 anti-mouse CD3e Antibody (clone 145-2C11)	BioLegend	100369; RRID: AB_2734149
TotalSeq™-A0095 Rat IgG2b, κ Isotype Ctrl Antibody (clone RTK4530)	BioLegend	400673
TotalSeq™-A0097 anti-mouse CD25 Antibody (clone PC61)	BioLegend	102055; RRID: AB_2749982
TotalSeq™-A0098 anti-mouse CD135 Antibody (clone A2F10)	BioLegend	135316; RRID: AB_2749983
TotalSeq™-A0103 anti-mouse/human CD45R/B220 Antibody	BioLegend	103263; RRID: AB_2734158
TotalSeq™-A0104 anti-mouse CD102 Antibody (clone 3C4 (MIC2/4))	BioLegend	105613; RRID: AB_2734167
TotalSeq™-A0105 anti-mouse CD115 (CSF-1R) Antibody (clone AFS98)	BioLegend	135533; RRID: AB_2734198
TotalSeq™-A0106 anti-mouse CD11c Antibody (clone N418)	BioLegend	117355; RRID: AB_2750352
TotalSeq™-A0107 anti-mouse CD21/CD35 (CR2/CR1) Antibody (clone 7E9)	BioLegend	123427; RRID: AB_2750540
TotalSeq™-A0108 anti-mouse CD23 Antibody (clone B3B4)	BioLegend	101635; RRID: AB_2750358
TotalSeq™-A0109 anti-mouse CD16/32 Antibody (clone 93)	BioLegend	101343; RRID: AB_2750532
TotalSeq™-A0110 anti-mouse CD43 Antibody (clone S11)	BioLegend	143211; RRID: AB_2750541
TotalSeq™-A0111 anti-mouse CD5 Antibody (clone 53-7.3)	BioLegend	100637; RRID: AB_2749985
TotalSeq™-A0112 anti-mouse CD62L Antibody (clone MEL-14)	BioLegend	104451; RRID: AB_2750364
TotalSeq™-A0113 anti-mouse CD93 (AA4.1, early B lineage) Antibody (clone AA4.1)	BioLegend	136513; RRID: AB_2750375
TotalSeq™-A0114 anti-mouse F4/80 Antibody (clone BM8)	BioLegend	123153; RRID: AB_2749986
TotalSeq™-A0115 anti-mouse FcεRIα Antibody (clone MAR-1)	BioLegend	134333; RRID: AB_2749987
TotalSeq™-A0117 anti-mouse I-A/I-E Antibody (clone M5/114.15.2)	BioLegend	107653; RRID: AB_2750505
TotalSeq™-A0118 anti-mouse NK-1.1 Antibody (clone PK136)	BioLegend	108755; RRID: AB_2750536
TotalSeq™-A0119 anti-mouse Siglec H Antibody (clone 551)	BioLegend	129615; RRID: AB_2750537
TotalSeq™-A0130 anti-mouse Ly-6A/E (Sca-1) Antibody (clone D7)	BioLegend	108147; RRID: AB_2750535
TotalSeq™-A0171 anti-human/mouse/rat CD278 (ICOS) Antibody (clone C398.4A)	BioLegend	313555; RRID: AB_2800824
TotalSeq™-A0173 anti-mouse CD206 (MMR) Antibody (clone C068C2)	BioLegend	141735
TotalSeq™-A0184 anti-mouse CD335 (NKp46) Antibody (clone 29A1.4)	BioLegend	137633; RRID: AB_2734199
TotalSeq™-A0190 anti-mouse CD274 (B7-H1, PD-L1) Antibody (clone MIH6)	BioLegend	153604; RRID: AB_2783125
TotalSeq™-A0191 anti-mouse/rat/human CD27 Antibody (clone LG.3A10)	BioLegend	124235; RRID: AB_2750344
TotalSeq™-A0192 anti-mouse CD20 Antibody (clone SA275A11)	BioLegend	150423; RRID: AB_2734214
TotalSeq™-A0193 anti-mouse CD357 (GITR) Antibody (clone DTA-1)	BioLegend	126319; RRID: AB_2734195
TotalSeq™-A0194 anti-mouse CD137 Antibody (clone 17B5)	BioLegend	106111; RRID: AB_2783048
TotalSeq™-A0195 anti-mouse CD134 (OX-40) Antibody (clone OX-86)	BioLegend	119426; RRID: AB_2750376
TotalSeq™-A0197 anti-mouse CD69 Antibody (clone H1.2F3)	BioLegend	104546; RRID: AB_2750539
TotalSeq™-A0198 anti-mouse CD127 (IL-7Rα) Antibody (clone A7R34)	BioLegend	135045; RRID: AB_2750009
TotalSeq™-A0200 anti-mouse CD86 Antibody (clone GL-1)	BioLegend	105047; RRID: AB_2750348
TotalSeq™-A0201 anti-mouse CD103 Antibody (clone 2E7)	BioLegend	121437; RRID: AB_2750349
TotalSeq™-A0202 anti-mouse CD64 (FcγRI) Antibody (clone X54-5/7.1)	BioLegend	139325; RRID: AB_2750367
TotalSeq™-A0203 anti-mouse CD150 (SLAM) Antibody (clone TC15-12F12.2)	BioLegend	115945; RRID: AB_2783055
TotalSeq™-A0204 anti-mouse CD28 Antibody (clone 37.51)	BioLegend	102129
TotalSeq™-A0212 anti-mouse CD24 Antibody (clone M1/69)	BioLegend	101841; RRID: AB_2750380
TotalSeq™-A0214 anti-human/mouse integrin β7 Antibody (clone FIB504)	BioLegend	321227; RRID: AB_2750504
TotalSeq™-A0225 anti-mouse CD196 (CCR6) Antibody (clone 29-2L17)	BioLegend	129825; RRID: AB_2783083
TotalSeq™-A0226 anti-mouse CD106 Antibody (clone 429 (MVCAM.A))	BioLegend	105725; RRID: AB_2783044
TotalSeq™-A0227 anti-mouse CD122 (IL-2Rb) Antibody (clone 5H4)	BioLegend	105909
TotalSeq™-A0228 anti-mouse CD183 (CXCR3) Antibody (clone CXCR3-173)	BioLegend	126543
TotalSeq™-A0229 anti-mouse CD62P (P-selectin) Antibody (clone RMP-1)	BioLegend	N/A
TotalSeq™-A0230 anti-mouse CD8b (Ly-3) Antibody (clone YTS156.7.7)	BioLegend	126623; RRID: AB_2800615
TotalSeq™-A0232 anti-mouse MAdCAM-1 Antibody (clone MECA-367)	BioLegend	120713; RRID: AB_2783058
TotalSeq™-A0236 Rat IgG1, κ Isotype Ctrl Antibody (clone RTK2071)	BioLegend	400459
TotalSeq™-A0237 Rat IgG1, λ Isotype Ctrl Antibody (clone G0114F7)	BioLegend	401919
TotalSeq™-A0238 Rat IgG2a, κ Isotype Ctrl Antibody (clone RTK2758)	BioLegend	400571
TotalSeq™-A0240 Purified Rat IgG2c, κ Isotype Ctrl Antibody (clone RTK4174)	BioLegend	400739
TotalSeq™-A0241 Armenian Hamster IgG Isotype Ctrl Antibody (clone HTK888)	BioLegend	400973
TotalSeq™-A0250 anti-mouse/human KLRG1 (MAFA) Antibody (clone 2F1/KLRG1)	BioLegend	138431; RRID: AB_2800648
TotalSeq™-A0376 anti-mouse CD195 (CCR5) Antibody (clone HM-CCR5)	BioLegend	107019; RRID: AB_2783049
TotalSeq™-A0377 anti-mouse CD197 (CCR7) Antibody (clone 4B12)	BioLegend	120129
TotalSeq™-A0378 anti-mouse CD223 (LAG-3) Antibody (clone C9B7W)	BioLegend	125229; RRID: AB_2783078
TotalSeq™-A0379 anti-mouse CD62E (E-selectin) Antibody (clone RME-1/CD62E)	BioLegend	N/A
TotalSeq™-A0381 anti-mouse Panendothelial Cell Antigen Antibody (clone MECA-32)	BioLegend	120507; RRID: AB_2783057
TotalSeq™-A0388 anti-mouse CD152 Antibody (clone UC10-4B9)	BioLegend	106325
TotalSeq™-A0415 anti-P2RY12 Antibody (clone S16007D)	BioLegend	848009; RRID: AB_2783419
TotalSeq™-A0416 anti-mouse CD300LG (Nepmucin) Antibody (clone ZAQ5)	BioLegend	147105; RRID: AB_2783116
TotalSeq™-A0422 anti-mouse CD172a (SIRPα) Antibody (clone P84)	BioLegend	144033; RRID: AB_2800670
TotalSeq™-A0424 anti-mouse CD14 Antibody (clone Sa14-2)	BioLegend	123333; RRID: AB_2800591
TotalSeq™-A0426 anti-mouse CD192 (CCR2) Antibody (clone SA203G11)	BioLegend	150625; RRID: AB_2783122
TotalSeq™-A0437 anti-mouse/human CD207 Antibody (clone 4C7)	BioLegend	N/A
TotalSeq™-A0438 anti-mouse/rat KKC2 Antibody	BioLegend	N/A
TotalSeq™-A0440 anti-mouse CD169 (Siglec-1) Antibody (clone N1/12)	BioLegend	142425; RRID: AB_2783106
TotalSeq™-A0441 anti-mouse CD71 Antibody (clone 3D6.112)	BioLegend	113824; RRID: AB_2800574
TotalSeq™-A0442 anti-mouse Notch 1 Antibody (HMN1-12)	BioLegend	130617; RRID: AB_2783085
TotalSeq™-A0443 anti-mouse CD41 Antibody (clone MWReg30)	BioLegend	133937; RRID: AB_2800635
TotalSeq™-A0448 anti-mouse CD204 (Msr1) Antibody (clone 1F8C33)	BioLegend	154703; RRID: AB_2783126
TotalSeq™-A0449 anti-mouse CD326 (Ep-CAM) Antibody (clone G8.8)	BioLegend	118237; RRID: AB_2800586
TotalSeq™-A0551 anti-mouse CD301a (MGL1) Antibody (clone LOM-8.7)	BioLegend	145611; RRID: AB_2783114
TotalSeq™-A0552 anti-mouse CD304 (Neuropilin-1) Antibody (clone 3E12)	BioLegend	145215; RRID: AB_2750383
TotalSeq™-A0554 anti-mouse CD309 (VEGFR2, Flk-1) Antibody (clone 89B3A5)	BioLegend	121921; RRID: AB_2783066
TotalSeq™-A0555 anti-mouse CD36 Antibody (clone HM36)	BioLegend	102621; RRID: AB_2800557
TotalSeq™-A0556 anti-mouse CD370 (CLEC9A-DNGR1) Antibody (clone 7H11)	BioLegend	N/A
TotalSeq™-A0557 anti-mouse CD38 Antibody (clone 90)	BioLegend	102733; RRID: AB_2750556
TotalSeq™-A0558 anti-mouse CD55 (DAF) Antibody (clone RIKO-3)	BioLegend	131809; RRID: AB_2783086
TotalSeq™-A0559 anti-mouse CD63 Antibody (clone NVG-2)	BioLegend	143915; RRID: AB_2783109
TotalSeq™-A0560 anti-mouse CD68 Antibody (clone FA-11)	BioLegend	137031; RRID: AB_2783099
TotalSeq™-A0561 anti-mouse CD79b (Igβ) Antibody (clone HM79-12)	BioLegend	132811; RRID: AB_2783087
TotalSeq™-A0562 anti-mouse CD83 Antibody (clone Michel-19)	BioLegend	121519; RRID: AB_2783061
TotalSeq™-A0563 anti-mouse CX3CR1 Antibody (clone SA011F11)	BioLegend	149041; RRID: AB_2783121
TotalSeq™-A0564 anti-mouse Folate Receptor β (FR-β) Antibody (clone 10/FR2)	BioLegend	153307; RRID: AB_2800690
TotalSeq™-A0565 anti-mouse MERTK (Mer) Antibody (clone 2B10C42)	BioLegend	151511
TotalSeq™-A0566 anti-mouse CD301b (MGL2) Antibody (clone URA-1)	BioLegend	146817; RRID: AB_2783115
TotalSeq™-A0567 anti-mouse Tim-4 Antibody (clone RMT4-54)	BioLegend	130011; RRID: AB_2783084
TotalSeq™-A0568 anti-mouse/rat XCR1 Antibody (clone ZET)	BioLegend	148227; RRID: AB_2783120
TotalSeq™-A0570 anti-mouse/rat CD29 Antibody (clone HMβ1-1)	BioLegend	102233; RRID: AB_2783042
TotalSeq™-A0573 anti-mouse CD140a Antibody (clone APA5)	BioLegend	135917; RRID: AB_2783094
TotalSeq™-A0595 anti-mouse CD11a Antibody (clone M17/4)	BioLegend	101125; RRID: AB_2783036
TotalSeq™-A0596 anti-mouse ESAM Antibody (clone 1G8/ESAM)	BioLegend	136209; RRID: AB_2800642

**Chemicals, Peptides, and Recombinant Proteins**		

Acetonnitrile	Sigma-Aldrich	14261
Antigenfix	Diapath	P0014
β-mercaptoethanol	Sigma-Aldrich	M3148
Bodipy 493/503	Thermo Fisher	D3922
Brefeldin	BioLegend	420601
BUV805 - Streptavidin	BD Horizon	564923
BV605 - Streptavidin	BD Horizon	563260
Calcium chloride dihydrate	Merck	1023821000
Collagenase A	Sigma-Aldrich	11088793001
CSF1fc	Bio-Rad	PPP031
D-(-)-Fructose	Merck-Millipore	1040071000
D-(+)-Glucose	Sigma-Aldrich	G7528
D(+)-Saccharose	VWR International	PROL27483.294
DAPI	Invitrogen	D1306; RIDD: AB_2629482
DMEM	Invitrogen	41965-039
Dnase I	Sigma-Aldrich	04 536 282 001
Donkey Serum	Abcam	ab7475
EDTA	Westburg	51234
EGTA	Sigma-Aldrich	E3889
Eosin	VWR International	MERC 1.15935
FcBlock 2.4G2	Bioceros	N/A
FCS	Bodinco	5010
Fixable Viability due Live/Dead - eFluor506	eBioscience	65-0866-18
Fixable Viability due Live/Dead - eFluor780	eBioscience	65-0865-18
Fixation/Permeabilization Solution Kit	BD Cytofix/Cytoperm	554714
FoxP3 Transcription factor staining buffer kit	eBioscience	00-5523-00
Gentamicin	GIBCO	15710-049
GlutaMAX	Thermo Fisher	35050-038
Gluteraldehyde 25%	Sigma-Aldrich	G5882
Goat Serum	Sigma-Aldrich	G9023
HCS LipidTOX™ Deep Red	Invitrogen	H34477
Hematoxylin	VWR International	MERC1.05174
HEPES	Sigma-Aldrich	H3375
Isopropanol	Vel	T0108
Methanol	Merck Millipore	13680502
Monensin	BioLegend	420701
Paraformaldehyde 10%	EMS	15712
PE-Cy5 - Streptavidin	BioLegend	405205
Phalloidin - Alexa Fluor™ 680	Invitrogen	A22286
Phenol Red	Sigma-Aldrich	P3532
Poly-Lysine	Sigma-Aldrich	P8920
Potassium chloride	Sigma-Aldrich	P9541
Potassium Ferricyanide	EMS	20150
ProLong Diamond	Thermo Fisher	P36970
Rat Serum	Sigma-Aldrich	R9759
RPMI 1640	GIBCO	52400-025
Saponin	Sigma-Aldrich	4521
Sodium bicarbonate	Sigma-Aldrich	792519
Sodium chloride	Sigma-Aldrich	746398
Sodium Dihydrogen Phosphate Monohydrate	Sigma-Aldrich	1063461000
Sodium phosphate dibasic dihydrate	Sigma-Aldrich	71643
Tissue-Tek O.C.T	Sakura Finetek	4583
Xylene	Prolabo	PROL28973.363

**Critical Commercial Assays**		

ALLin HS Red Taq Mastermix 2x	highQu	HQ.HSM0350
Cholesterol/Cholesteryl Ester Quantitation Kit	Abnova	KA0829
PicroSirius Red Staining Kit	Abcam	ab150681
PrimeFlow RNA assay kit	Thermo Fisher	88-18005-210
RNEasy Plus Micro Kit	QIAGEN	74034
SensiFAST cDNA Synthesis Kit	Bioline	BIO-65054
SensiFAST SYBR No-ROX Kit	Bioline	BIO-98020
*Spp1* Alexa Fluor 647 probes Type 1 for PrimeFlow	Thermo Fisher	PF-204
Triglyceride Colorimetric Assay Kit	Cayman	10010303
Ultra Sensitive Mouse Insulin ELISA kit	Crystal Chem Inc.	90080

**Oligonucleotides**		

*Actb -* qPCR FWD	IDT	GCTTCTAGGCGGACTGTTACTGA
*Actb -* qPCR REV	IDT	GCCATGCCAATGTTGTCTCTTAT
*Atf3* - qPCR FWD	IDT	GAGGATTTTGCTAACCTGACACC
*Atf3* - qPCR REV	IDT	TTGACGGTAACTGACTCCAGC
*Bmp10 -* qPCR FWD	IDT	ACCAGACGTTGGCAAAAGTCAGGC
*Bmp10 -* qPCR REV	IDT	GATGATCCAGGAGTCCCACCCAAT
*Bmp2* - qPCR FWD	IDT	TGCACCAAGATGAACACAGC
*Bmp2* - qPCR REV	IDT	GTGCCACGATCCAGTCATTC
*Bmp6* - qPCR FWD	IDT	AAGACCCGGTGGTGGCTCTA
*Bmp6* - qPCR REV	IDT	CTGTGTGAGCTGCCCTTGCT
*Calr -* qPCR FWD	IDT	GAATACAAGGGCGAGTGGAA
*Calr -* qPCR REV	IDT	GGGGGAGTATTCAGGGTTGT
*Cd36 -* qPCR FWD	IDT	GGAGCCATCTTTGAGCCTTCA
*Cd36 -* qPCR REV	IDT	GAACCAAACTGAGGAATGGATCT
*Col1a2 -* qPCR FWD	IDT	TGAAGTGGGTCTTCCAGGTCTTTC
*Col1a2 -* qPCR REV	IDT	CACCCTTGTTACCGGATTCTCCTT
*Cp* - qPCR FWD	IDT	TCTACCAAGGAGTAGCCAGGA
*Cp* - qPCR REV	IDT	ATCTTCCCTCTCATCCGTGC
*Csf1 -* qPCR FWD	IDT	CGGGCATCATCCTAGTCTTGCTGACTGT
*Csf1 -* qPCR REV	IDT	ATAGTGGCAGTATGTGG GGGGCATCCTC
*Cxcl10 -* qPCR FWD	IDT	GTCTGAGTGGGACTCAAGGGAT
*Cxcl10 -* qPCR REV	IDT	TCAACACGTGGGCAGGATAG
*Cyp2e1* - qPCR FWD	IDT	GTTGCCTTGCTTGTCTGGAT
*Cyp2e1* - qPCR REV	IDT	AGGAATTGGGAAAGGTCCTG
*Dll4 -* qPCR FWD	IDT	TTCCAGGCAACCTTCTCCGA
*Dll4 -* qPCR REV	IDT	ACTGCCGCTATTCTTGTCCC
*Fabp4* - qPCR FWD	IDT	GATGAAATCACCGCAGACGACA
*Fabp4* - qPCR REV	IDT	ATTGTGGTCGACTTTCCATCCC
*Fga -* qPCR FWD	IDT	TTCTGCTCTGATGATGACTGGAA
*Fga -* qPCR REV	IDT	GGCTTCGTCAATCAACCCTTT
*Gdf2 -* qPCR FWD	IDT	TGAGTCCCATCTCCATCCTC
*Gdf2 -* qPCR REV	IDT	ACCCACCAGACACAAGAAGG
*Hspa5* - qPCR FWD	IDT	ACCCACCAAGAAGTCTCAGATCTT
*Hspa5* - qPCR REV	IDT	CGTTCACCTTCATAGACCTTGATTG
*Il1b* - qPCR FWD	IDT	GCAACTGTTCCTGAACTCAACT
*Il1b* - qPCR REV	IDT	ATCTTTTGGGGTCCGTCAACT
*Il34* - qPCR FWD	IDT	CTTTGGGAAACGAGAATTTGGAGA
*Il34* - qPCR REV	IDT	GCAATCCTGTAGTTGATGGGGAAG
*Il6* - qPCR FWD	IDT	TGATGGATGCTACCAAACTGG
*Il6* - qPCR REV	IDT	TTCATGTACTCCAGGTAGCTATGG
*Lcn2 -* qPCR FWD	IDT	TGCCACTCCATCTTTCCTGTT
*Lcn2 -* qPCR REV	IDT	GGGAGTGCTGGCCAAATAAG
*Mki67 -* qPCR FWD	IDT	ATCATTGACCGCTCCTTTAGGT
*Mki67 -* qPCR REV	IDT	GCTCGCCTTGATGGTTCCT
*Tgfb1 -* qPCR FWD	IDT	CTCCCGTGGCTTCTAGTGC
*Tgfb1 -* qPCR REV	IDT	GCCTTAGTTTGGACAGGATCTG
*Tnf -* qPCR FWD	IDT	TCTTCTCATTCCTGCTTGTGG
*Tnf -* qPCR REV	IDT	GGTCTGGGCCATAGAACTGA
*Tnfaip2* - qPCR FWD	IDT	AAACCAATGGTGATGGAAACTG
*Tnfaip2* - qPCR REV	IDT	GTTGTCCCATTCGTCATTCC

**Software and Algorithms**		

Adobe Illustrator	Adobe	www.adobe.com
Enrichr	(Kuleshov et al., 2016)	http://amp.pharm.mssm.edu/Enrichr/
FlowJo v10.6.1	FlowJo	https://www.flowjo.com
GeneOntology	(Ashburner et al., 2000; The Gene Ontology Consortium, 2019)	http://geneontology.org/
GraphPad Prism 8	GraphPad	https://www.graphpad.com/
Harmony	(Korsunsky et al., 2019)	https://www.github.com/immunogenomics/harmony
Ilastik	(Berg et al., 2019)	https://www.ilastik.org/
ImageJ	(Schneider et al., 2012)	https://imagej.nih.gov/ij
Single-Cell Signature Explorer algorithm	(Pont et al., 2019)	https://sites.google.com/site/fredsoftwares/products/single-cell-signature-explorer
FastCAR	m.berg@umcg.nl	https://github.com/MarijnBerg/FastCAR
WEB-based GEne SeT AnaLysis Toolkit	(Liao et al., 2019)	http://www.webgestalt.org/
Zen Black	ZEISS Microscopy	www.zeiss.com

**Experimental Models**		

Mouse: B6(C)-Ccr2 ^tm1.1Cln/J^	The Jackson Laboratory	JAX: 027619
Mouse: C57BL/6j SPF	Janvier Labs	N/A
Mouse: B6-Clec4f^hDTR/YFP-CIPHE^	(Scott et al., 2016)	N/A

### Lead Contact

Further information and requests for resources and reagents should be directed to and will be fulfilled by the lead contact, Charlotte Scott (charlotte.scott@irc.vib-ugent.be).

#### Materials Availability

This study did not generate new unique reagents.

#### Data and Code Availability

The datasets generated during this study have been deposited in the Gene Expression Omnibus public database under accession number GEO: GSE156059.

#### Additional Resources

The scRNA-seq and CITE-seq datasets can be visualized, analyzed and downloaded at www.livercellatlas.org

### Experimental Model and Subject Details

#### *In Vivo* Animal Studies

The following mice were used in this study; C57BL/6J (Janvier), Ccr2GFP/+ (Satpathy et al., 2013) and *Clec4f*-DTR (Scott et al., 2016). All mice were used on a C57BL/6 background and only male mice were used for diet experiments while a mix of male and female mice were used for all experiments performed with normal chow diet. Mice were put on the diets from 5 weeks of age and sacrificed at indicated time points. All other mice were used between 6 and 12 weeks of age. All mice were bred and/or maintained at the VIB (Ghent University) under specific pathogen free conditions. All animals were randomly allocated to experimental groups. All experiments were performed in accordance with the ethical committee of the Faculty of Science, UGent and VIB.

### Method Details

#### Diet induced MAFLD

To induce MAFLD and NASH, mice were fed a western diet (WD) high in fat, sugar and cholesterol as described previously (Ganz et al., 2015). This consisted of 58% fat, 1% cholesterol (Research Diets; D09061703i) and drinking water was supplemented with 23.1g/L fructose (MPBio) and 18.9g/L sucrose (VWR). Control mice were fed a standard diet with 11kcal% fat with corn starch (D12328i; Research Diets).

#### Intraperitoneal Glucose Tolerance Test

Following overnight fastening, mice were administered 2g/kg glucose via intraperitoneal injection. Blood glucose concentrations were measured 0, 15, 30, 60, 120 and 180 min later.

#### Insulin ELISA

Following overnight fasting, blood was collected from the tail, serum was collected and fasting insulin were measured on undiluted serum according to the manufacturer’s instructions using the Ultra Sensitive Mouse Insulin ELISA kit (Crystal Chem)

#### Colorimetric assays

After sacrifice, mice were bled by cardiac puncture and serum was collected. Serum cholesterol & cholesteryl esters were measured according to the manufacturer’s instructions using the Cholesterol & Cholesteryl Ester Quantitation Kit (Abnova). Serum triglycerides were measured according to the manufacturer’s instructions using the Triglyceride Colorimetric Assay Kit (Cayman).

#### Histological analysis

Livers were removed rapidly, a piece taken and fixed immediately in fresh 4% paraformaldehyde at RT for 24 h. Fixed livers were then dehydrated and embedded in paraffin, after which 5 μm cross sections were obtained using a Microm HM360. Sections were then deparaffinized, rehydrated and stained with hematoxylin and eosin using standard protocols. Sections were also stained for Picro-Sirius Red according to the manufacturer’s instructions using the Picro Sirius Red Stain kit (Abcam). Sections were imaged using a Zeiss AxioScan.Z1.

#### Isolation of Liver Cells

Liver cells were isolated by liver perfusion and digestion as described previously (Bonnardel et al., 2019). Briefly, after retrograde cannulation, livers were perfused for 1-2mins with an EGTA-containing solution, followed by a 5min (6ml/min) perfusion with 0.2mg/mL collagenase A. Livers were then removed, minced and incubated for 20mins with 0.4mg/mL collagenase A and 10U/mL DNase at 37°C. All subsequent procedures were performed at 4°C. Samples were filtered over a 100 μm mesh filter and red blood cells were lysed. Samples were again filtered over a 40 μm mesh filter. After two centrifugation steps of 1 min at 50 g to isolate hepatocytes, remaining liver cells (leukocytes, LSECs and HSCs) were centrifuged at 400 g for 5mins before proceeding to antibody staining for flow cytometry.

#### Flow Cytometry and Cell Sorting

0.5-5x10^6^ cells were pre-incubated with 2.4G2 antibody (Bioceros) to block Fc receptors and stained with appropriate antibodies at 4°C in the dark for 30-45 min. Cell viability was assessed using Fixable Viability dyes (eFluor780 or eFluor506; Thermo Fisher) and cell suspensions were analyzed with a BD FACSymphony or purified using a BD FACSAria II or III and FlowJo software (BD). Bodipy staining (Thermo Fisher) was performed after surface antibody staining for 15 min at room temperature. The full list of antibodies used can be found in the Key Resource Table. The Primeflow assay (Thermo Fisher) for Spp1 expression was performed in 96-well U-bottom plates according to the manufacturer’s instructions. Ki67 and Tbet staining was performed by fixing and permeabilizing extracellularly stained cells according to the manufacturer’s instructions using the FoxP3 Transcription factor staining buffer Kit (eBioscience). Lipidtox staining was performed after fixing and permeabilizing extracellularly stained cells according to the manufacturer’s instructions using the Fixation/Permeabilization Solution Kit (BD Cytofix/Cytoperm). To measure intracellular cytokines, 0.5-5x10^6^ cells were incubated for 3.5 h at 37°C in DMEM with 10%FCS, 1% glutamax, 0.1% β-mercaptoethanol, 0.5% gentamicin with 1X monensin and 1X brefeldin A (Biolegend). After incubation, cells were stained extracellularly as above. Intracellular cytokine staining was performed by fixing and permeabilizing extracellularly stained cells according to the manufacturer’s instructions using the Fixation/Permeabilization Solution Kit (BD Cytofix/Cytoperm).

#### Confocal microscopy

Confocal staining was performed as described previously (Bonnardel et al., 2019). Immediately after sacrificing mice with CO_2_, inferior vena cava were cannulated and livers were perfused (4 mL/min) with Antigenfix (Diapath) for 5 min at room temperature. After excision, 2-3 mm slices of livers were fixed further by immersion in Antigenfix for 1 h at 4°C, washed in PBS, infused overnight in 34% sucrose and frozen in Tissue-Tek OCT compound (Sakura Finetek). 20 μm-thick slices cut on a cryostat (Microm HM 560, Thermo Scientific) were rehydrated in PBS for 5 min, permeabilized with 0.5% saponin and non-specific binding sites were blocked for 30 min with 2% bovine serum albumin, 1% fetal calf serum and 1% donkey or goat serum for 30 min. Tissue sections were labeled overnight at 4°C with primary antibodies followed by incubation for 1 h at room temperature with secondary antibodies. When two rat antibodies were used on the same section, the directly conjugated rat antibody was incubated for 1 h after staining with the unconjugated and anti-rat secondary and after an additional blocking step with 1% rat serum for 30 min. Slides were mounted in ProLong Diamond, imaged with a Zeiss LSM780 confocal microscope (Carl Zeiss, Oberkochen, Germany) with spectral detector and using spectral unmixing and analyzed using ImageJ and QuPath software.

#### Generation of BM chimeras

Bone marrow chimeras were generated as described previously (Scott et al., 2016). Briefly, 5 week old male C57BL/6J wild-type mice (CD45.2) on normal chow were anaesthetized by intraperitoneal administration of Ketamine (150 mg/kg) and Xylazine (10 mg/kg). Mice were lethally irradiated with 8 Gy, with the livers being protected with a 3-cm-thick lead cover. Once recovered from the anesthesia, mice were reconstituted by intravenous administration of 5-10 × 10^6^ BM cells from CD45.1/CD45.2 wild-type mice. Mice were placed on either the SD or WD immediately after reconstitution. Chimerism was assessed 18 or 24 weeks after irradiation by flow cytometry.

#### CSF1Fc administration

Mice received the SD or WD for 24 weeks, before being injected subcutaneously with 1 mg/kg CSF1Fc or PBS for 4 days. Mice were sacrificed on day 6 and the % and number of KCs was assessed by flow cytometry.

#### DT administration

*Clec4f*-DTR mice or WT littermate controls were injected intraperitoneally with 500ng DT. Mice were sacrificed on 3 or 6 days after DT administration.

#### RNA Sequencing, CITE-seq and qPCR

##### Sorting and RNA Isolation

40000-160000 live CD45^+^ cells or CD45^-^ cells from livers of a C57BL/6 mouse fed the SD or WD for 12, 24 and 36 weeks were purified, centrifuged at 400 g for 5 min and stained with 2.4G2 antibody to block Fc receptors and CITE-seq antibodies for 20mins at 4°C, before being washed in excess PBS with 2% FCS and 2mM EDTA. Cells were then resuspended in PBS with 0.04% BSA at ∼1000 cells/mL. Cell suspensions (target recovery of 8000-10000 cells) were loaded on a GemCode Single-Cell Instrument (10x Genomics, Pleasanton, CA, USA) to generate single-cell Gel Bead-in-Emulsions (GEMs). ScRNA-Seq libraries were prepared using GemCode Single-Cell 3ʹGel Bead and Library Kit (10x Genomics, V3 technology) according to the manufacturer’s instructions. Briefly, GEM-RT was performed in a 96-Deep Well Reaction Module: 55°C for 45min, 85°C for 5 min; end at 4°C. After RT, GEMs were broken down and the cDNA was cleaned up with DynaBeads MyOne Silane Beads (Thermo Fisher Scientific, 37002D) and SPRIselect Reagent Kit (SPRI; Beckman Coulter; B23318). cDNA was amplified with 96-Deep Well Reaction Module: 98°C for 3 min; cycled 12 times: 98°C for 15 s, 67°C for 20 s, and 72°C for 1 min; 72°C for 1 min; end at 4°C. Amplified cDNA product was cleaned up with SPRIselect Reagent Kit prior to enzymatic fragmentation. Indexed sequencing libraries were generated using the reagents in the GemCode Single-Cell 3ʹ Library Kit with the following intermediates: (1) end repair; (2) A-tailing; (3) adaptor ligation; (4) post-ligation SPRIselect cleanup and (5) sample index PCR. Pre-fragmentation and post-sample index PCR samples were analyzed using the Agilent 2100 Bioanalyzer.

##### qPCR

RNA was extracted from 25000 sorted macrophages (CLEC4F^+^ TIM4^+^, CLEC4F^+^ TIM4^-^ or CLEC4F^-^ TIM4^-^) or from 5000-25000 Hepatocytes, LSECs, HSCs or cholangiocytes from livers of C57BL/6 mice fed the SD or WD for 12, 24 and 36 weeks using a RNeasy Plus micro kit (QIAGEN). Sensifast cDNA synthesis kit (Bioline) was used to transcribe total RNA to cDNA. Real-time RT-PCR using SensiFast SYBR No-Rox kit (Bioline) was performed to determine gene expression, therefore a PCR amplification and detection instrument LightCycler 480 (Roche) was used. Gene expression was normalized to β-actin gene expression.

##### Bulk RNA Sequencing Analysis

Libraries were constructed using the Illumina TruSeq RNA Preparation Kit. RNA sequencing was performed at the VIB Nucleomics Core using Illumina NextSeq500 with these parameters: High Output v2.5, 75 bp, Single Reads (76-8-8-0), 1.4 pM + 1.73% PhiX. All samples passed quality control based on the results of FastQC (v0.11.9). Reads were mapped to the mouse reference genome (mm10) via HiSat2 (v2.2.0) with max-intronlen set to 1000000. The aligned reads were counted via FeatureCounts (v2.0.0). The R package limma (v3.42.2) was used to normalize the data and to perform differential expression analysis. Genes that didn’t have a count per million (cpm) value > 1 in at least 5 samples were removed. As such, we continued with 13421 genes. Benjamini-Hochberg was used to adjust the p values for multiple testing. The DE genes were defined based on a log2-ratio > 1 or < −1 and adj.P value < 0.05. For the heatmap we first transformed the normalized expression table as ‘log2(2ˆexpTable + 1)’ and subsequently scaled the values per gene by calculating the mean expression per gene and then subtracting that mean value of each expression value. The R package triwise (v0.99.5) was used to create the triwise plots.

##### scRNA-Sequencing Analysis

Sequencing libraries were loaded on an Illumina HiSeq with sequencing settings recommended by 10X Genomics (26/8/0/98 – 2.1pM loading concentration, ADT and cDNA libraries were pooled in a 25:75 ratio). Sequencing was performed at the VIB Nucleomics Core (VIB, Leuven). The demultiplexing of the raw data was performed using CellRanger software (10x – version 3.1.0; cellranger mkfastq which wraps Illumina’s bcl2fastq). The reads obtained from the demultiplexing were used as the input for ‘cellranger count’ (CellRanger software), which aligns the reads to the mouse reference genome (mm10) using STAR and collapses to unique molecular identifier (UMI) counts. The result is a large digital expression matrix with cell barcodes as rows and gene identities as columns. The aggregation of the samples was performed using ‘cellranger aggr’ (CellRanger software).

#### Preprocessing Data

Preprocessing of the data was done by the scater R package (v1.12.2) according to workflow proposed by the Marioni lab (Lun et al., 2016). Outlier cells were identified based on 3 metrics (library size, number of expressed genes and mitochondrial proportion) and cells were first tagged as outliers when they were x median absolute deviation (MADs) higher or lower than the median value of each metric. For library size 4 MADs was used, for the number of expressed genes 4 MADs for used and for the mitochondrial proportion 5 MADs was used. For the patient data were respectively 3 MADs, 3 MADs and 5 MADs used. As second filtering, a principal component analysis plot was generated using the runPCA function of the scater R package (default parameters). Outlier cells in this principal component analysis plot were identified using the R package mvoutlier. For the CD45^-^ cells, due to inclusion of hepatocytes an additional cleanup was necessary to remove ambient mRNA signals. Samples were corrected for ambient RNA using the R package FastCAR (v0.1.0). We defined an empty droplet as a cell barcode with at least 10 UMIs and less than a certain cut off. To define this cut off we first made a plot showing on the y axis how many UMIs are present in a barcode and on the x-as the number of barcodes (same plot as in the CellRanger web_summary output). By making this plot, a clear ‘drop’ in UMI counts becomes visible and we put our cut off just before this ‘drop’. This cut off is always lower than the minimal UMI count from the ‘filtered’ CellRanger output. The corrected counts were merged together using the R package Harmony (v1.0). For all samples, after these initial analysis steps, additional low quality (low UMI counts, > 8% of mitochondrial genes), contaminating (potential doublets) and actively proliferating cells were also removed from the analysis. Low-abundance genes were removed by removing all genes that weren’t expressed (count > 0) in at least 3 cells. The counts were normalized and log2 transformed via the NormalizeData function of the Seurat R package (v3.1.1) using default parameters. Detecting highly variable genes, finding clusters and creating UMAP plots was done using the Seurat pipeline. For the clustering, the first 20 principal components were used and 0.6 was used as the resolution parameter. The monocyte-macrophage UMAP was generated starting from the counts and using specific cells from the full UMAP (based on Mafb, Ly6c2, Fcgr1 and Adgre1 expression, 21156 cells). Then the same Seurat pipeline was followed as described above. The first 20 PCs were used and 0.8 was the resolution parameter. Differential gene expression was assessed using the findMarker function of the Seurat pipeline.

For the CITE-Seq data, we first checked in how many cells each antibody was expressed. The lowest expressed antibody was expressed in only 1 cell and was hence excluded from further analysis, while the second lowest antibody was expressed in 41% of the cells. The expression data were processed using the Seurat workflow, with CLR normalization and scaling of the data performed using the default parameters. Marker Enrichment Modeling (Diggins et al., 2017) was performed on the CITE-Seq data using the MEM R package (v2.0.0). The MEM heatmap was generated using pheatmap (v1.0.12). For each antibody we checked per cluster if the MEM values were similar to those of the isotype control antibodies. When MEM values of an antibody are ± 0.5 compared with the MEM values of the isotype across all clusters, the antibody was considered to not give a real signal above background and was hence removed from further analyses. The plots showing the expression of specific antibodies (Figure 1B) were generated by collating all cells with a count > 0 and calculating the 98% quantile cut-off of counts, with all the cells above this value being colored in red.

#### Metabolomic profiling of Macrophages

ResKCs (10x10^4^ – 25x10^4^) were sorted in DMEM supplemented with 10% FCS (Bodinco) using a BD FACSARIA II and ARIA III. Cells were then incubated for 30 min at 37°C, before being pelleted at −9°C and washed in 1ml ice cold NaCl (0.9%). Cells were then pelleted at −9°C and snap frozen in liquid nitrogen. Samples were than extracted in 100uL of 30:50:20 acetonitrile:methanol:milli-Q H_2_O pre-cooled at −20°C, dried using speed vac and resuspended in 20uL of 30:50:20 acetonitrile:methanol:milli-Q H_2_O for metabolites that are identified in negative ionization mode (polar), or in H_2_O for metabolites that are identified in positive ionization mode (non-polar). Metabolite quantification was carried out using Agilent 1290 Infinity II UHPLC in line with a Bruker impact II QTOF-MS operated in full scan (MS1) mode. LC separation was performed on a Waters CSH-C18 column (100 × 2.1 mm, 1.7 μm particles) using a binary solvent gradient of 100% buffer A (0.1% formic acid in water) to 97% buffer B (50:50 methanol:acetonitrile). Flow rate was 400 μL/min, autosampler temperature was 4°C, and injection volume was 3 μL. Data processing was performed using TASQ™ Software (Target Analysis for Screening and Quantification) (Bruker Daltonics Inc.).

#### Generation of M0, M1 and M2 *in vitro* macrophages

Bone marrow cells were isolated from tibias and femurs of WT mice and grown in complete medium (RPMI-1640 medium containing 10 mM glucose, 2 mM L-glutamine, 100 U ml^–1^ penicillin-streptomycin and 10% FCS) with 20 ng ml^–1^ murine macrophage colony- stimulating factor 1 (CSF-1; Peprotech) for 7 days, and supplemented with CSF-1 on days 3 and 5. On day 7 macrophages were harvested and then maintained in 20 ng ml^–1^ CSF-1 for subsequent experiments in which they were either maintained in medium alone (M0), or stimulated with 50 ng ml^–1^ IFN-γ (R&D systems) and 20 ng ml^-1^ LPS (M1), or 20 ng ml^–1^ IL-4 (M2) (Peprotech) for 18 h.

#### Lipidomic profiling of Macrophages

The protocol for lipid extraction was adapted from Matyash et al. (Matyash et al., 2008). Frozen cell pellets (60000-150000 cells) were resuspended in ice cold PBS and transferred to glass tubes before the addition of methanol and methyl tert-butyl ether. The tubes were then shaken for 1 h at 4°C. Water was added to separate the phases before centrifugation at 1,000 x g for 10 min. The upper organic phase was collected and dried in a Genevac EZ2 speed vac. Samples were resuspended in 2:1:1 isopropanol:acetonitrile:milli-Q H2O prior to analysis. LC-MS was carried out using an Agilent Zorbax Eclipse Plus C18 column using an Agilent 1290 Infinity II UHPLC inline with an Agilent 6495 Triple Quad QQQ-MS. Lipids were identified by fragmentation and retention time, and were quantified using Agilent Mass Hunter software.

#### HCS LipidTOX Deep Red staining for microscopy

25000 ResKCs were sorted into DMEM supplemented with 10% FCS (Bodinco) using an ARIA II and ARIA III (BD Biosciences). Cells were incubated on coverslips pre-coated with poly-lysine for 30 min at 37°C. Cells were washed and then fixed using Antigenfix (Diapath). Cells were washed and stained with DAPI (Invitrogen) and LipidTOX Deep Red staining (Invitrogen) for 1 h at room temperature. Slides were mounted in ProLong Diamond, imaged with a Zeiss LSM780 confocal microscope (Carl Zeiss, Oberkochen, Germany) and analyzed using ImageJ software.

#### FIB-SEM

100000 CLEC4F^+^ KCs were sorted into DMEM supplemented with 10% FCS (Bodinco) using a BD FACSARIA II and ARIA III. Cells were resuspended in DMEM +10% FCS at room temperature and were incubated for 2 h at 37°C to adhere onto coverslips. Following that, samples were incubated in freshly prepared fixative (2% paraformaldehyde (PFA, Applichem), 2.5% glutaraldehyde (GA, EMS) in 0.1M sodium cacodylate (Sigma-Aldrich) buffer, pH7.4) at RT for 30 min. Fixative was removed by washing in 0.1M cacodylate buffer and samples were incubated in 1% osmium (OsO4, EMS), 1.5% potassium ferrocyanide (Sigma-Aldrich) in 0.1M cacodylate buffer for 40 min at RT. After washing in ddH2O, samples were incubated overnight at 4°C in 1:3 UAR in H_2_O (Uranyl Acetate Replacement, EMS). The next day, UAR was removed by washing in ddH2O. After final washing steps the samples were dehydrated using solutions of increasing EtOH concentration (30%, 50%, 70%, 90%, 2x 100%) for 3 min each. Subsequent infiltration with resin (Spurr’s, EMS) was done by first incubating in 50% resin in ethanol for 2 h, followed by at least 3 changes of fresh 100% resin (including 1 overnight incubation). Next, samples were embedded in fresh resin and cured in the oven at 65°C for 72 h.

For FIB-SEM imaging, embedded cells were mounted on aluminum SEM stubs (diameter 12 mm) using conductive epoxy (Circuit Works) and samples were coated with ∼20nm of Platinum (Quorum Q150T ES). FIB-SEM imaging was performed using a Zeiss Crossbeam 540 system with Atlas5 software. The Focused Ion Beam (FIB) was set to remove 5nm sections by propelling gallium ions at the surface. Imaging was performed at 1.5kV and 1nA using an ESB (back-scattered electron) detector.

#### Lipid droplet analysis

The FIB-SEM data was segmented using deep convolutional neural networks, specifically U-Net (Ronneberger et al., 2015). In total, 2292 patches of size 256x256 were selected across 8 samples. The pixels in these patches that correspond to lipid droplets were labeled by an expert. The obtained labeled dataset was split in 66/34% train/test data. For training the network, we employed weighted cross entropy minimization (weight selected to balance classes) batch normalization, 16 initial feature maps (i.e., 4 times smaller as in (Ronneberger et al., 2015)) and standard data augmentations (small scaling, rotations, flips, random deformations and noise perturbations). A threshold is then applied to the final probability maps to obtain a segmentation of the droplets. This segmentation was further cleaned by removing noise components (objects smaller than 3x3x3 = 27 voxels) and applying a morphological opening with a spherical structure element (radius of 2 voxels). This final segmentation allowed us to extract the total volume of the droplets. The neural network was implemented in PyTorch and post-processing was performed using MATLAB. The code is publicly available on www.github.com/jorisroels/lipid-droplets.

#### Quantification of Microscopy

To detect the Macrophages, a custom groovy script (used with the eleventh milestone release on the path to QuPath v0.2.0) (Bankhead et al., 2017) has been written using the F4/80 marker, and then cells have been classified based on their CLEC4F, TIM4 and F4/80 expression. The script splits the image into tiles, extracts the F4/80 channel image, applies a median filter to remove the noise, applies a threshold to keep only the positive signal object and uses the Analyze Particles from ImageJ to filter the smallest particle by area. The ROIs (Region of Interest) are sent to Qupath as annotation, the adjacent annotations due to the tiling are merged back and holes within the annotations are filled and converted to cell detections. Script is available upon request.

Feature extraction was performed using QuPath and detected cells classified using QuPath based on their CLEC4F, TIM4 and F4/80 expression. Identification of Desmin^hi^ areas was performed using QuPath: based on pixel intensity from the Desmin channel, a pixel classifier was trained, the high/low Desmin Areas detected and the number of each cell type was then counted in Desmin^hi^ and Desmin^lo^ areas. Large vessels were identified manually based on expression of Desmin and CD31 and these regions were excluded from the analysis.

### Quantification and Statistical Analysis

In all experiments, data are presented as mean ± SEM and/or individual data points are presented unless stated otherwise. Statistical tests were selected based on appropriate assumptions with respect to data distribution and variance characteristics. Student’s t test (two-tailed) was used for the statistical analysis of differences between two groups. One-way ANOVA with Bonferroni's multiple comparisons test was used for the statistical analysis of differences between more than two groups. Details of the precise test used for each analysis can be found in the figure legends. Statistical significance was defined as p < 0.05. Sample sizes were chosen according to standard guidelines. Number of animals is indicated as ‘‘n’’. The investigators were not blinded to the mouse group allocation.

## Supplemental Information

Document S1. Figures S1–S7

Table S1. DEGs per Cluster and per Cell Type (SD versus WD) in Total Live CD45^+^ Cells, Related to Figure 1

Table S2. DEGs per Cluster and per Cell Type (SD versus WD) in Total Live CD45^-^ Cells, Related to Figure 3

Table S3. DEGs per Cluster in Monocyte/Macrophage Cells, Related to Figures 4 and S5

Table S4. DEGs and Signatures from Different Comparisons of Monocyte/Macrophage scRNA-seq Analysis, Related to Figures 4 and S5

Table S5. Signatures of TIM4+ ResKCs, TIM4- moKCs, and CLEC4F- Macs from Bulk RNA-seq, Related to Figure S6

Table S6. DEGs between Mac Subsets at Different Time Points on SD and WD in scRNA-seq and Bulk RNA-seq Analysis, Related to Figures 5, 6, and 7

Table S7. DEGs and Signatures of Adipose Tissue and Hepatic LAMs, Related to Figure 6

Document S2. Article plus Supplemental Information
