# Supplementary material for: Osteopontin Expression Identifies a Subset of Recruited Macrophages Distinct from Kupffer Cells in the Fatty Liver
Source: Immunity. 2020 Sep 15;53(3):641–657.e14. doi: 10.1016/j.immuni.2020.08.004 (PMC7501731; doi:10.1016/j.immuni.2020.08.004)
Supplement: Supplementary file 1 [file mmc1.pdf]

Immunity, Volume *53*

Supplemental Information

Osteopontin Expression Identifies a Subset of Recruited Macrophages Distinct from Kupffer Cells in the Fatty Liver

Anneleen Remmerie, Liesbet Martens, Tinne Thoné, Angela Castoldi, Ruth Seurinck, Benjamin Pavie, Joris Roels, Bavo Vanneste, Sofie De Prijck, Mathias Vanhockerhout, Mushida Binte Abdul Latib, Lindsey Devisscher, Anne Hoorens, Johnny Bonnardel, Niels Vandamme, Anna Kremer, Peter Borghgraef, Hans Van Vlierberghe, Saskia Lippens, Edward Pearce, Yvan Saeys, and Charlotte L. Scott

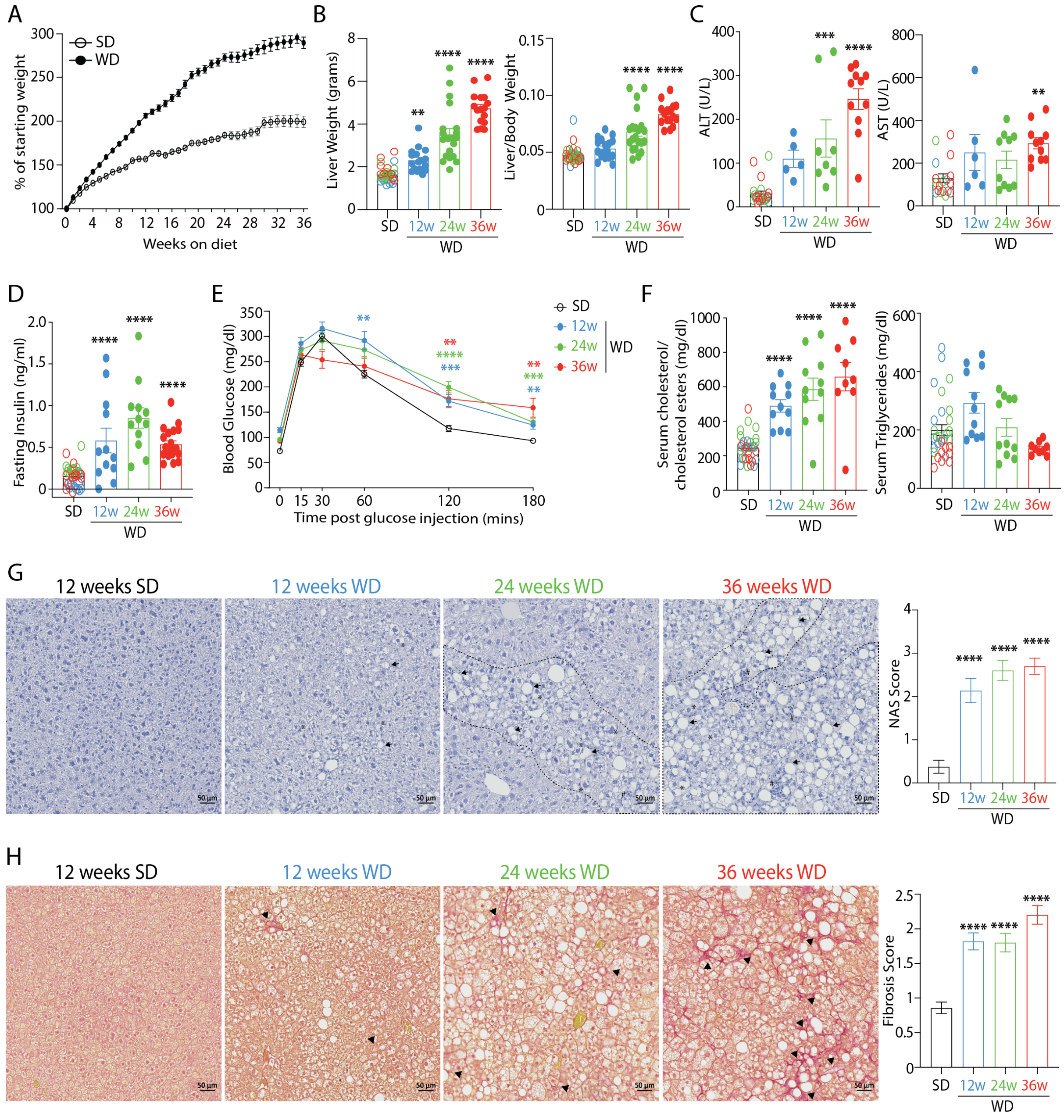

Figure S1: Feeding mice a Western diet induces features of human MAFLD; related to Figure 1.

C57BL/6 mice were fed either a standard diet (SD) or Western diet (WD) for 12, 24 or 36 weeks. (A) Body weights shown as a percentage of the starting weight of mice on the appropriate diet. n=11-37 per timepoint. (B) Liver weight and liver-to-body weight ratio from mice fed the diets for 12 (blue), 24 (green) or 36 (red) weeks. Open circles show SD, closed circles show WD. Data are pooled from 2-4 independent experiments, with n=9-21. ****p<0.0001 One-way ANOVA compared with pooled SD. (C) Serum ALT and AST in mice on SD or WD at indicated time points. Data are pooled from 2 independent experiments with n=4-11. **p<0.01, ***p<0.001, ****p<0.0001 One-way ANOVA compared with pooled SD. (D)

Insulin resistance was determined by measuring fasting insulin at the indicated time points on SD or WD. Data are pooled from 2 independent experiments with n=12-20. ****p<0.0001 One-way ANOVA compared with pooled SD. (E) Intraperitoneal glucose tolerance test in mice fed the SD (all times pooled - black, open circles) or WD for 12 (blue), 24 (green) or 36 (red) weeks. Data are pooled from 2 independent experiments with n=12-28. **p<0.01, ***p<0.001, ****p<0.0001 Two-way ANOVA compared with pooled SD. (F) Serum cholesterol and cholesteryl esters (left) and serum triglycerides (right) measured in mice at indicated time points on SD or WD. Data are pooled from 2 independent experiments with n=9-11. ****p<0.0001 One-way ANOVA compared with pooled SD. (G,H) Histological appearance and scoring of livers as assessed by (G) H&E staining, with dashed lines indicating zones with more established steatosis (indicated with arrow), hepatocyte ballooning (indicated with *) and inflammatory infiltrates (indicated with #). (H) Sirius Red staining, with arrow heads indicating fibrosis as assessed by deposition of collagen. Images are representative of 9-11 mice per timepoint per diet. NAS and fibrosis scores are calculated from each mouse. ****p<0.0001 One-way ANOVA compared with pooled SD. All error bars indicate ±SEM.

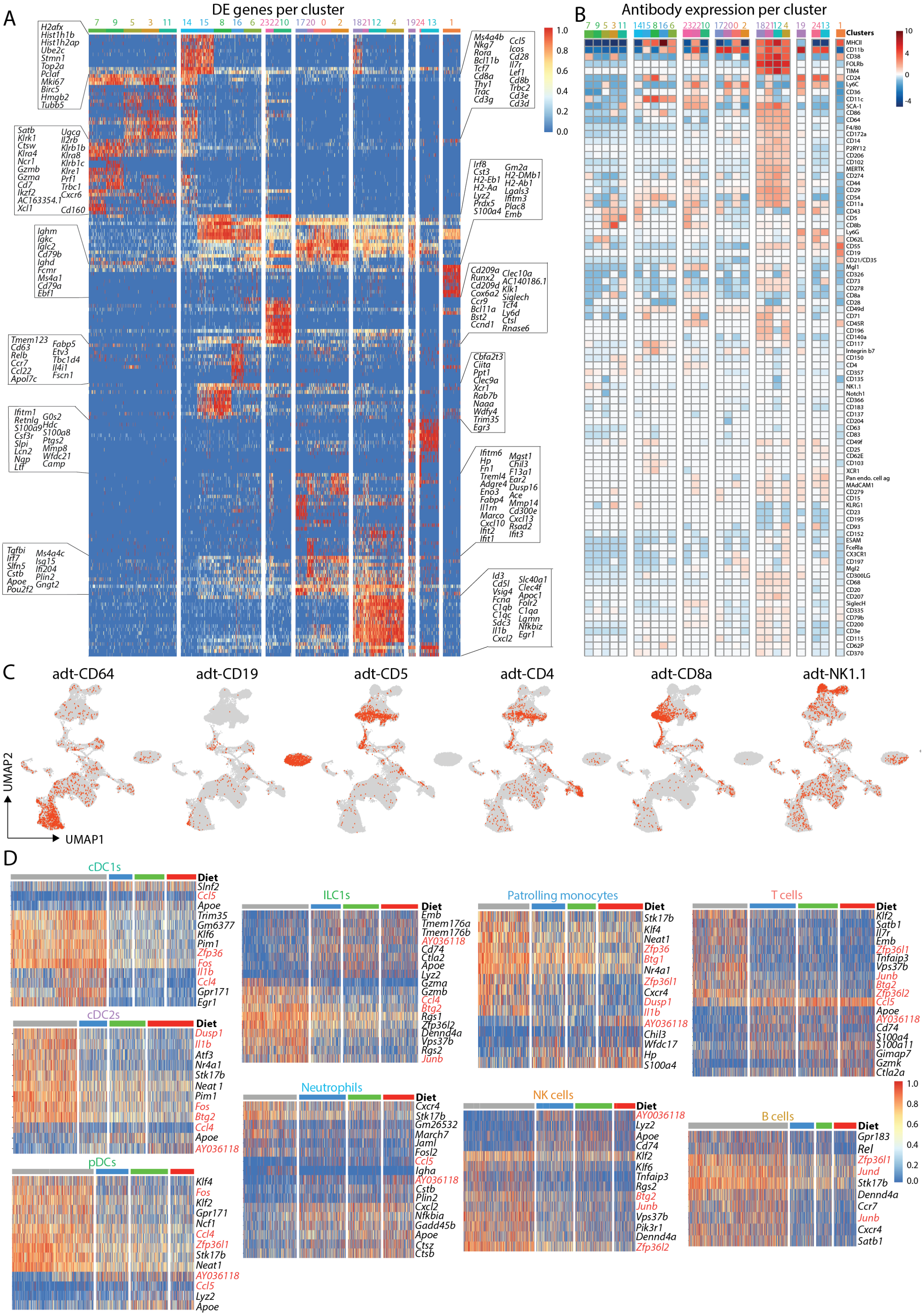
A
DE genes per cluster
B
Antibody expression per cluster
Clusters
C
adt-CD64
adt-CD19
adt-CD5
adt-CD4
adt-CD8a
adt-NK1.1
UMAP2
UMAP1
D
cDC1s
cDC2s
pDCs
ILC1s
Patrolling monocytes
T cells
Neutrophils
NK cells
B cells
Diet

Figure S2: Identification of distinct immune populations in MAFLD liver; related to Figure 1.

Total live $CD45^+$ cells were sorted from livers of mice fed the SD or WD for 12, 24 or 36 weeks, (1 mouse per timepoint per diet) stained with total-seq A antibodies and loaded onto the 10X Chromium platform. After QC and exclusion of doublets, 56407 cells remained. (A) Heatmap showing top 10 distinguishing genes per cluster. (B) Marker Enrichment Modelling (MEM) heatmap showing enriched protein expression per cluster determined using CITE-seq after removal of antibodies with expression profiles similar to appropriate isotype controls. (C) UMAP showing expression of indicated proteins based on antibody mapping of each cluster. (D) Heatmaps showing top DEGs for the indicated cell types as assessed by comparing SD and WD samples pooled from all timepoints. Genes in red are DEGs conserved in multiple cell types.

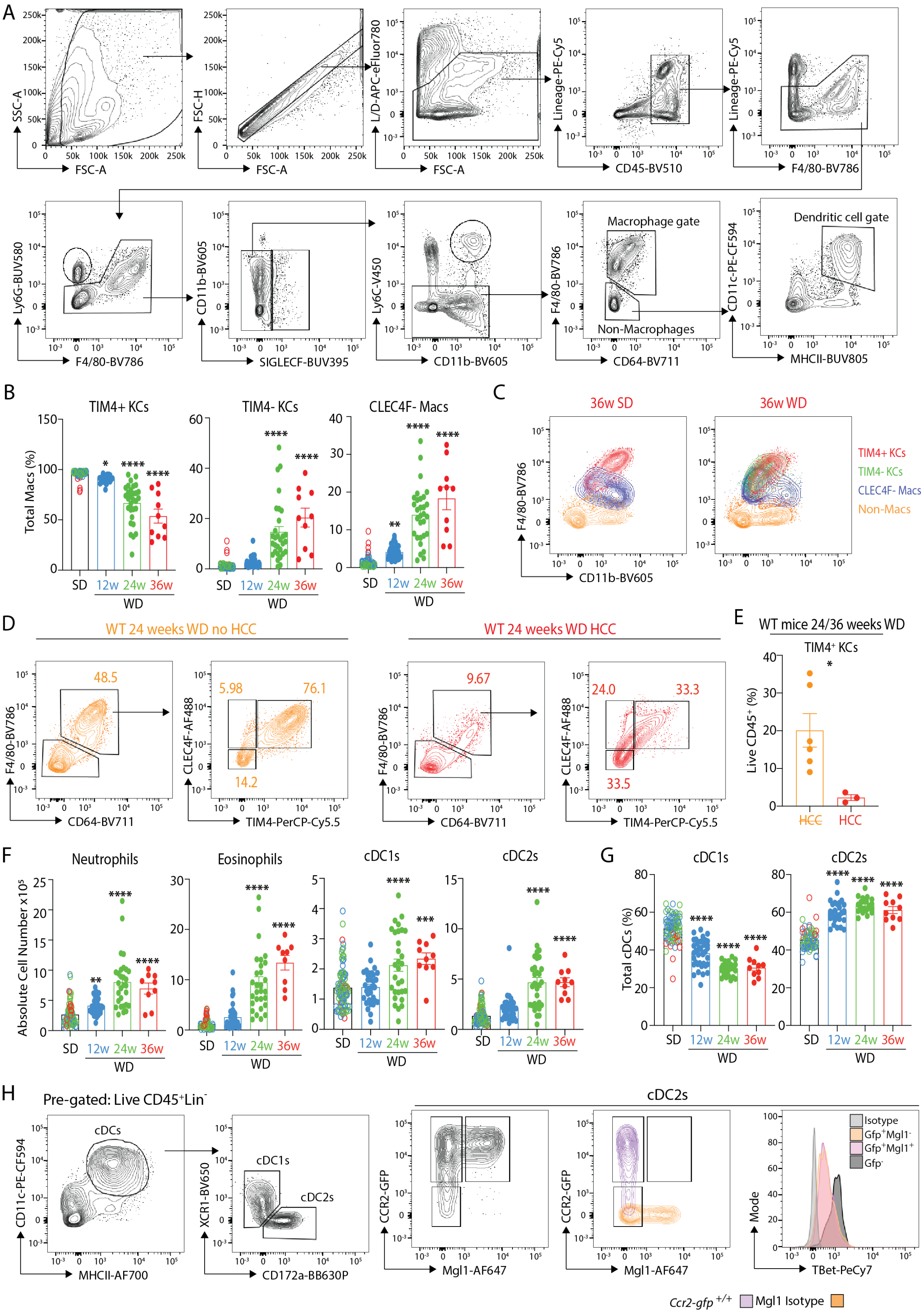
A
SSC-A
FSC-A
FSC-H
FSC-A
L/D-APC-eFluor780
FSC-A
Lineage-PE-Cy5
CD45-BV510
Lineage-PE-Cy5
F4/80-BV786
Ly6G-BUV580
F4/80-BV786
CD11b-BV605
SIGLECF-BUV395
Ly6C-V450
CD11b-BV605
F4/80-BV786
CD64-BV711
Macrophage gate
Non-Macrophages
CD11c-PE-CF594
MHCII-BUV805
Dendritic cell gate
B
TIM4+ KCs
TIM4- KCs
CLEC4F- Macs
Total Macs (%)
SD
12w
24w
36w
WD
C
36w SD
36w WD
F4/80-BV786
CD11b-BV605
TIM4+ KCs
TIM4- KCs
CLEC4F- Macs
Non-Macs
D
WT 24 weeks WD no HCC
WT 24 weeks WD HCC
48.5
5.98
76.1
14.2
9.67
24.0
33.3
33.5
F4/80-BV786
CD64-BV711
CLEC4F-AF488
TIM4-PerCP-Cy5.5
E
WT mice 24/36 weeks WD
TIM4⁺ KCs
Live CD45⁺ (%)
HCC
HCC
F
Neutrophils
Eosinophils
cDC1s
cDC2s
Absolute Cell Number x10⁵
G
cDC1s
cDC2s
Total cDCs (%)
H
Pre-gated: Live CD45⁺Lin⁻
cDCs
CD11c-PE-CF594
MHCII-AF700
cDC1s
cDC2s
XCR1-BV650
CD172a-BB630P
cDC2s
CCR2-GFP
Mgl1-AF647
Mode
TBet-PeCy7
Isotype
Gfp⁺Mgl1⁻
Gfp⁺Mgl1⁺
Gfp⁻
Ccr2-gfp ⁺/⁺
Mgl1 Isotype

Figure S3: Additional myeloid cells in MAFLD; related to Figure 2.

(A) Flow cytometry gating strategy to identify indicated myeloid cell populations. (B) % of indicated macrophage populations amongst total macs at indicated timepoints on SD or WD. Data are pooled from 3-7 independent experiments with n=9-38. *p<0.05, **p<0.01, ***p<0.001, ****p<0.0001 One-way ANOVA compared with pooled SD as a control. (C) Expression of F4/80 and CD11b by indicated macrophage populations and non-macrophages as gated in A. (D) Flow plots from mice with (red) or without (orange) spontaneous development of HCC after 24 weeks on WD. Numbers represent % of parent population. (E) $TIM4^+$ ResKCs as a % of total Live $CD45^+$ cells in mice with (red) or without (orange) spontaneous development of HCC after 24 or 36 weeks on WD. Mice without HCC are taken from the same experiments as the mice where spontaneous HCC was observed. *p<0.05 Student's t test. Data are pooled from 2 independent experiments with n=3-6. (F) Absolute cell numbers per liver of indicated cell types after 12 (blue), 24 (green) or 36 (red) weeks on diets as determined by flow cytometry. Open circles = SD, closed circles = WD. (G) % of cDC1s and cDC2s amongst total cDCs after different times on SD or WD. Data are pooled from 3-7 independent experiments with n=9-38. *p<0.05, **p<0.01, ***p<0.001, ****p<0.0001 One-way ANOVA compared with pooled SD as a control. (H) Flow cytometry gating showing heterogeneity of cDC2s based on expression of Mgl1, CCR2-GFP and Tbet in steady state *Ccr2-gfp*$^{Tg/+}$ mice. Data are representative of 3 mice. All error bars indicate ±SEM.

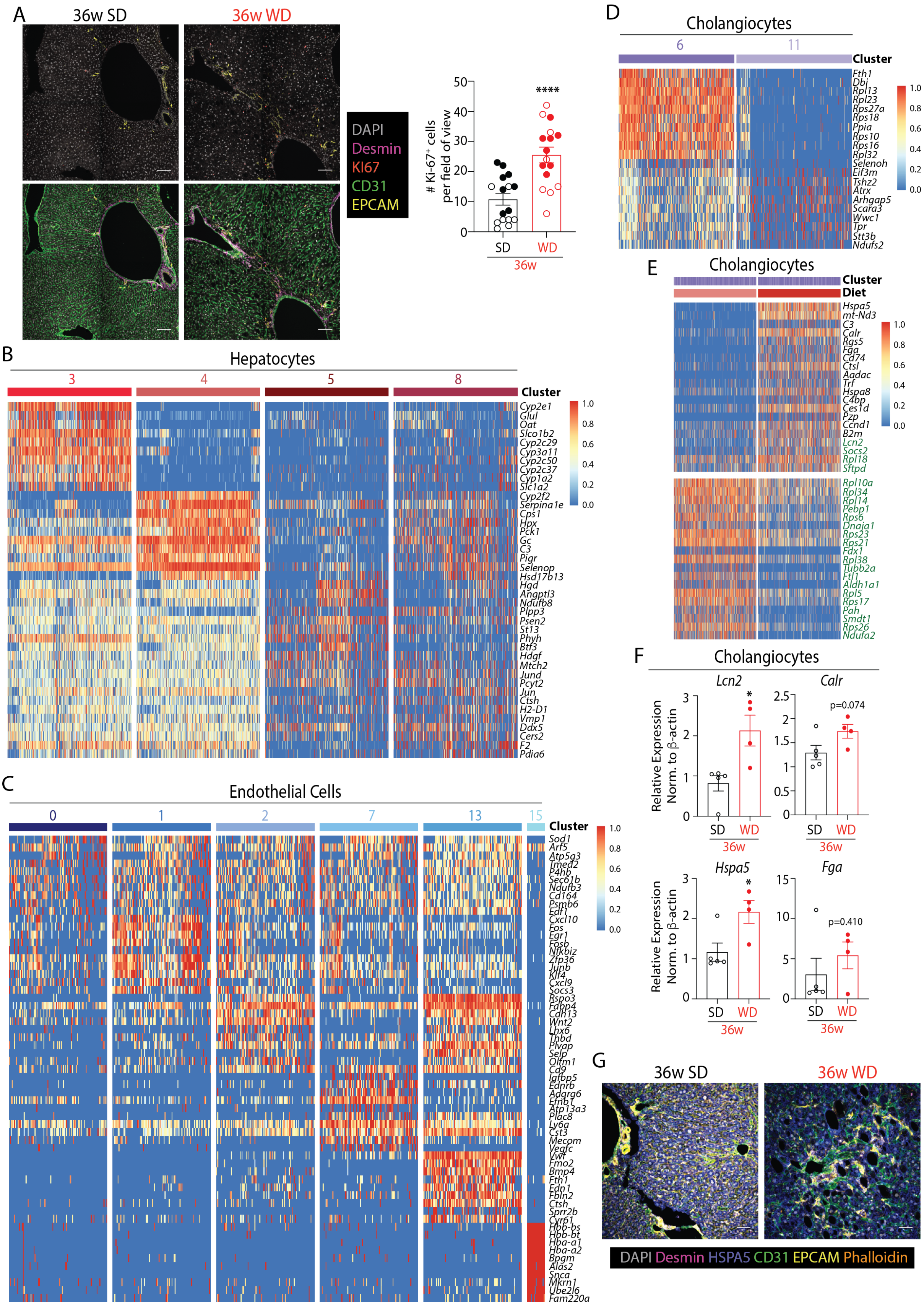
A
36w SD
36w WD
DAPI
Desmin
KI67
CD31
EPCAM
Ki-67⁺ cells per field of view
0
10
20
30
40
50
****
SD
WD
36w
B
Hepatocytes
3
4
5
8
Cluster
Cyp2e1
Glul
Oat
Slco1b2
Cyp2c29
Cyp3a11
Cyp2c50
Cyp2c37
Cyp1a2
Slc1a2
Cyp2f2
Serpina1e
Cps1
Hpx
Pck1
Gc
C3
Pigr
Selenop
Hsd17b13
Hgd
Angptl3
Ndufb8
Plpp3
Psen2
St13
Phyh
Btf3
Hdgf
Mtch2
Jund
Pcyt2
Jun
Ctsh
H2-D1
Vmp1
Ddx5
Cers2
F2
Pdia6
1.0
0.8
0.6
0.4
0.2
0.0
C
Endothelial Cells
0
1
2
7
13
15
Cluster
Sod1
Arf5
Atp5g3
Tmed2
P4hb
Sec61b
Ndufb3
Cd164
Psmb6
Edf1
Cxcl10
Fos
Egr1
Fosb
Nfkbiz
Zfp36
Junb
Klf4
Cxcl9
Socs3
Rspo3
Fabp4
Cdh13
Wnt2
Lhx6
Thbd
Plvap
Selp
Olfm1
Cd9
Igfbp5
Ednrb
Adgrg6
Efnb1
Atp13a3
Plac8
Ly6a
Cst3
Mecom
Vegfc
Vwf
Fmo2
Bmp4
Fth1
Edn1
Fbln2
Ctsh
Sprr2b
Cyr61
Hbb-bs
Hbb-bt
Hba-a1
Hba-a2
Bpgm
Alas2
Snca
Mkrn1
Ube2l6
Fam220a
1.0
0.8
0.6
0.4
0.2
0.0
D
Cholangiocytes
6
11
Cluster
Fth1
Dbi
Rpl13
Rpl23
Rps27a
Rps18
Ppia
Rps10
Rps16
Rpl32
Selenoh
Eif3m
Tshz2
Atrx
Arhgap5
Scaf11
Wwc1
Tpr
Stt3b
Ndufs2
1.0
0.8
0.6
0.4
0.2
0.0
E
Cholangiocytes
Cluster
Diet
Hspa5
mt-Nd3
C3
Calr
Rgs5
Fga
Cd74
Ctsl
Aadac
Trf
Hspa8
C4bp
Ces1d
Pzp
Ccnd1
B2m
Lcn2
Socs2
Rpl18
Sftpd
Rpl10a
Rpl34
Rpl14
Pebp1
Rps6
Dnaja1
Rps23
Rps21
Fdx1
Rpl38
Tubb2a
Ftl1
Aldh1a1
Rpl5
Rps17
Pah
Smdt1
Rps26
Ndufa2
1.0
0.8
0.6
0.4
0.2
0.0
F
Cholangiocytes
Lcn2
Calr
Hspa5
Fga
Relative Expression Norm. to β-actin
*
p=0.074
p=0.410
0
1
2
3
0.5
1.5
2.5
5
10
15
SD
WD
36w
G
36w SD
36w WD
DAPI Desmin HSPA5 CD31 EPCAM Phalloidin

Figure S4: CD45$^-$ cell responses in MAFLD; related to Figure 3.

(A) Confocal microscopy images showing expression of DAPI (grey), Ki67 (red) and EPCAM (yellow) (top panel) and CD31 (green) and Desmin (magenta) (bottom panel) in livers of mice fed the SD or WD for 36 weeks. Scale bar is 100um. Images shown represent 4 fields of view. Number of Ki-67$^+$ cell per field of view quantified from 8 fields of view (from 3 different liver slices and stains) per mouse with 2 mice per diet. Different mice are shown in filled or empty circles. ****p<0.0001 Student's t test. Error bars indicate ±SEM. (B) Heatmaps showing top DEGs in between the different clusters of hepatocytes identified (SD and WD pooled). (C) Heatmaps showing top DEGs in between the different clusters of endothelial cells identified (SD and WD pooled). (D) Heatmaps showing top DEGs in between the different clusters of cholangiocytes identified (SD and WD pooled). (E) Heatmaps showing top 20 up- and down-regulated DEGs in cholangiocytes between SD and WD fed mice (24 and 36 weeks). Genes in green represent those DEGs specifically altered at the 36 week timepoint. (F) qPCR analysis for indicated genes in cholangiocytes. Data are from a single experiment with n=4-5 per group. *p<0.05 Student's t test. Error bars indicate ±SEM. (G) Confocal microscopy showing expression of HSPA5 (blue), DAPI (grey), Desmin (magenta), CD31 (green), EPCAM (yellow) and Phalloidin (orange) in a mouse fed the SD or WD for 36 weeks. Data are representative of 2 mice per diet.

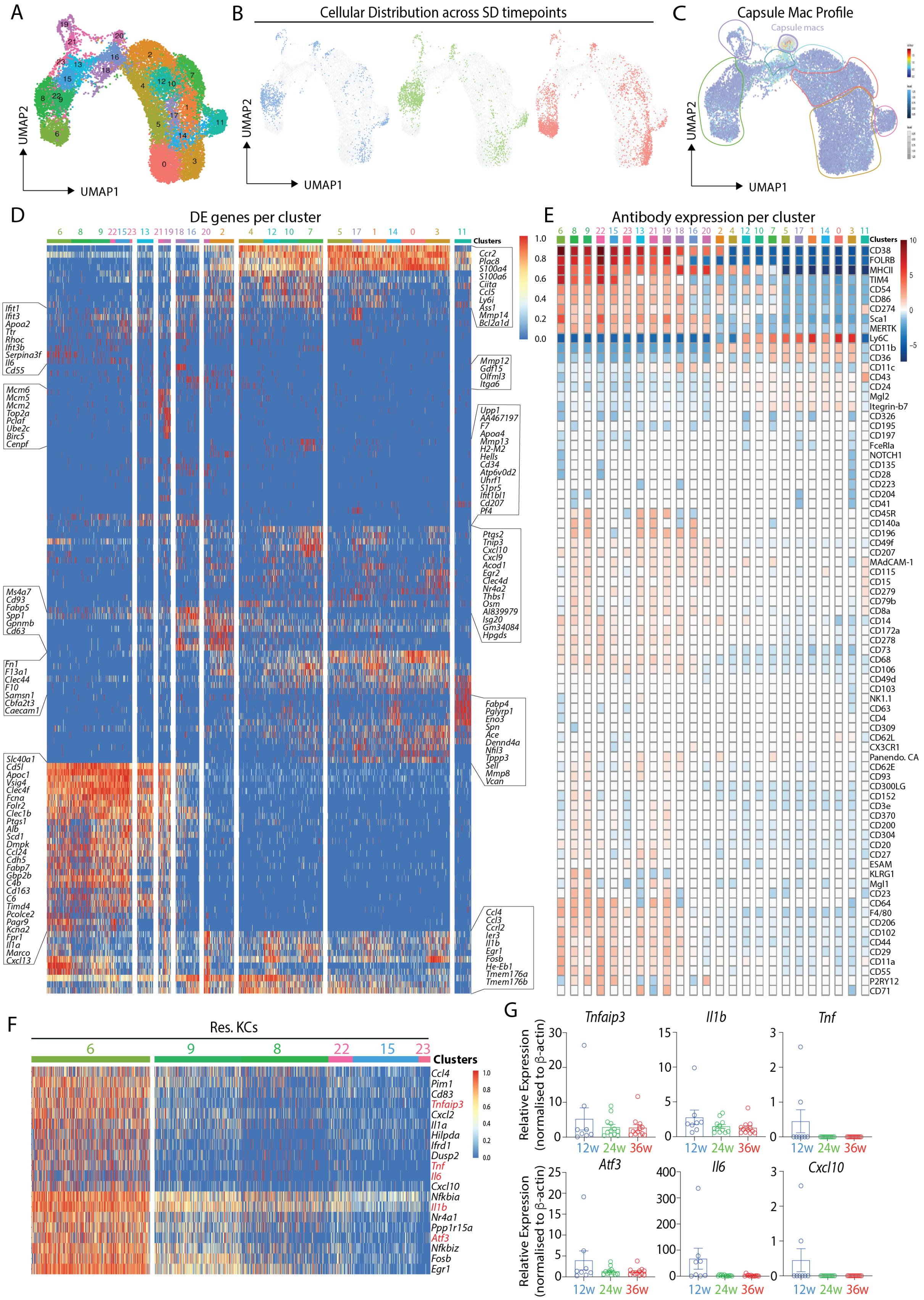
A
UMAP1
UMAP2
B
Cellular Distribution across SD timepoints
C
Capsule Mac Profile
Capsule macs
D
DE genes per cluster
Clusters
Ifit1 Ifit3 Apoa2 Ttr Rhoc Ifit3b Serpina3f Il6 Cd55
Mcm6 Mcm5 Mcm2 Top2a Pclaf Ube2c Birc5 Cenpf
Ms4a7 Cd93 Fabp5 Spp1 Gpnmb Cd63
Fn1 F13a1 Clec44 F10 Samsn1 Cbfa2t3 Caecam1
Slc40a1 Cd5l Apoc1 Vsig4 Clec4f Fcna Folr2 Clec1b Ptgs1 Alb Scd1 Dmpk Ccl24 Cdh5 Fabp7 Gbp2b C4b Cd163 C6 Timd4 Pcolce2 Pagr9 Kcna2 Fpr1 Il1a Marco Cxcl13
Ccr2 Plac8 S100a4 S100a6 Ciita Ccl5 Ly6i Ass1 Mmp14 Bcl2a1d
Mmp12 Gdf15 Olfml3 Itga6
Upp1 AA467197 F7 Apoa4 Mmp13 H2-M2 Hells Cd34 Atp6v0d2 Uhrf1 S1pr5 Ifit1bl1 Cd207 Pf4
Ptgs2 Tnip3 Cxcl10 Cxcl9 Acod1 Egr2 Clec4d Nr4a2 Thbs1 Osm AI839979 Isg20 Gm34084 Hpgds
Fabp4 Pglyrp1 Eno3 Spn Ace Dennd4a Nfil3 Tppp3 Sell Mmp8 Vcan
Ccl4 Ccl3 Ccrl2 Ier3 Il1b Egr1 Fosb He-Eb1 Tmem176a Tmem176b
E
Antibody expression per cluster
Clusters
CD38 FOLRB MHCII TIM4 CD54 CD86 CD274 Sca1 MERTK Ly6C CD11b CD36 CD11c CD43 CD24 Mgl2 Itegrin-b7 CD326 CD195 CD197 FceRIa NOTCH1 CD135 CD28 CD223 CD204 CD41 CD45R CD140a CD196 CD49f CD207 MAdCAM-1 CD115 CD15 CD279 CD79b CD8a CD14 CD172a CD278 CD73 CD68 CD106 CD49d CD103 NK1.1 CD63 CD4 CD309 CD62L CX3CR1 Panendo. CA CD62E CD93 CD300LG CD152 CD3e CD370 CD200 CD304 CD20 CD27 ESAM KLRG1 Mgl1 CD23 CD64 F4/80 CD206 CD102 CD44 CD29 CD11a CD55 P2RY12 CD71
F
Res. KCs
Clusters
Ccl4 Pim1 Cd83 Tnfaip3 Cxcl2 Il1a Hilpda Ifrd1 Dusp2 Tnf Il6 Cxcl10 Nfkbia Il1b Nr4a1 Ppp1r15a Atf3 Nfkbiz Fosb Egr1
G
Relative Expression (normalised to β-actin)
Tnfaip3
Il1b
Tnf
Atf3
Il6
Cxcl10
12w 24w 36w

Figure S5: Macrophage Heterogeneity in MAFLD; related to Figure 4.

(A) UMAP showing all clusters identified amongst cells of monocyte and macrophage lineage pooled from SD and WD fed mice at 12, 24 and 36 weeks (1 mouse per timepoint). (B) Distribution of cells on SD at indicated timepoints. (C) The capsule macrophage signature (Sierro et al., 2017) was mapped onto the liver mac UMAP to identify cells with a similar profile using the Signature Finder algorithm (Pont et al., 2019). (D) Heatmap showing top 5 DEGs in each cluster from UMAP in Fig. S4A with all cells from all diets and timepoints pooled. (E) MEM heatmap showing enriched expression of indicated surface proteins per cluster identified in monocyte/macrophage UMAP (Fig. S4A). (F) Heatmap showing top 20 DEGs between cluster 6 and other ResKC clusters (9,8,22,15 and 23). (G) qPCR analysis for indicated genes on sorted ResKCs from mice fed the SD for 12,24 or 36 weeks. Data are pooled from 2 independent experiments with n=8-12. Error bars indicate ±SEM.

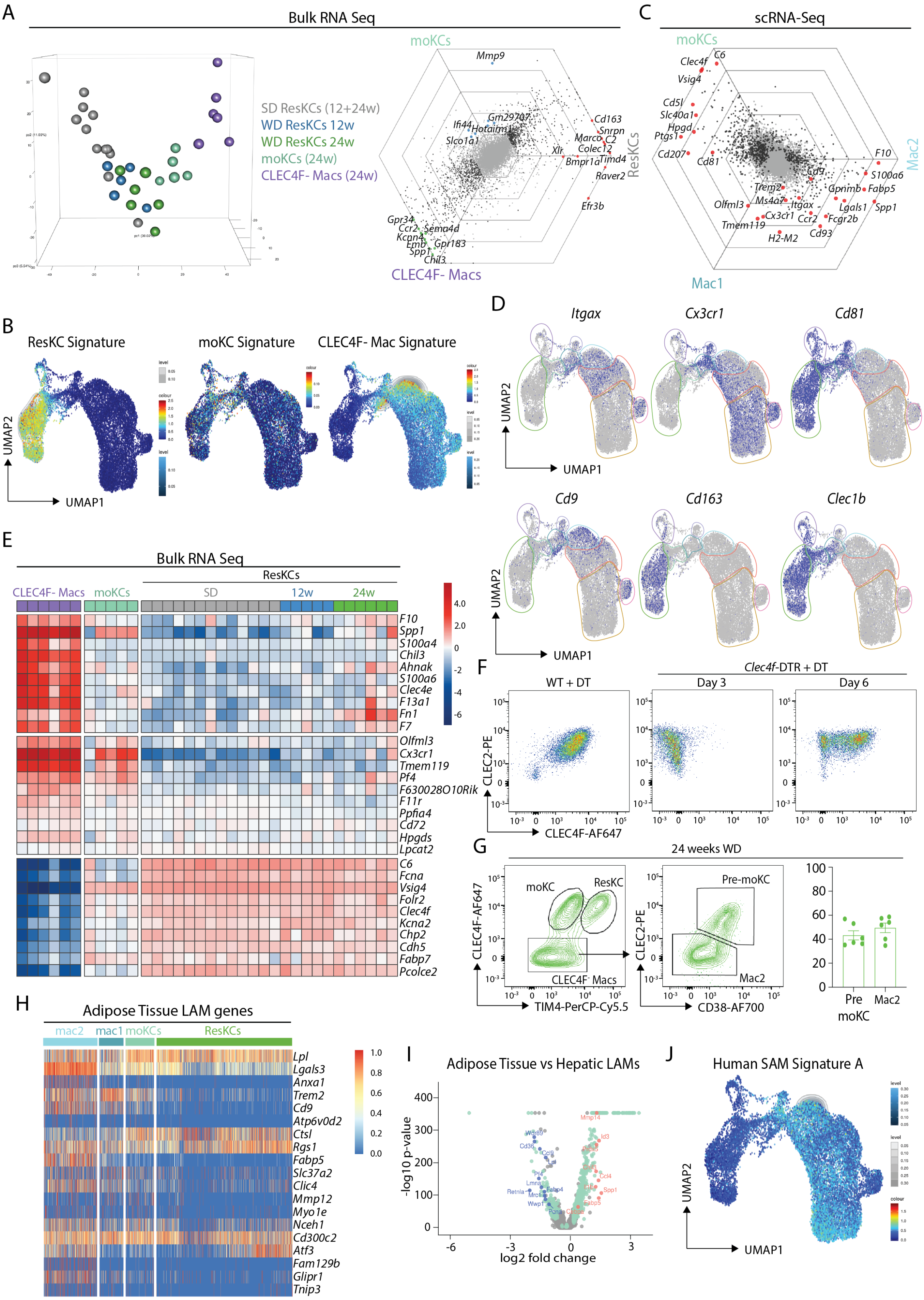

A
Bulk RNA Seq
SD ResKCs (12+24w)
WD ResKCs 12w
WD ResKCs 24w
moKCs (24w)
CLEC4F- Macs (24w)
moKCs
ResKCs
CLEC4F- Macs
C
scRNA-Seq
moKCs
Mac2
Mac1
B
ResKC Signature
moKC Signature
CLEC4F- Mac Signature
UMAP2
UMAP1
D
Itgax
Cx3cr1
Cd81
Cd9
Cd163
Clec1b
E
Bulk RNA Seq
ResKCs
CLEC4F- Macs
moKCs
SD
12w
24w
F10
Spp1
S100a4
Chil3
Ahnak
S100a6
Clec4e
F13a1
Fn1
F7
Olfml3
Cx3cr1
Tmem119
Pf4
F630028O10Rik
F11r
Ppfia4
Cd72
Hpgds
Lpcat2
C6
Fcna
Vsig4
Folr2
Clec4f
Kcna2
Chp2
Cdh5
Fabp7
Pcolce2
F
Clec4f-DTR + DT
WT + DT
Day 3
Day 6
CLEC2-PE
CLEC4F-AF647
G
24 weeks WD
moKC
ResKC
CLEC4F- Macs
Pre-moKC
Mac2
CLEC4F-AF647
TIM4-PerCP-Cy5.5
CLEC2-PE
CD38-AF700
Pre moKC
Mac2
H
Adipose Tissue LAM genes
mac2
mac1
moKCs
ResKCs
Lpl
Lgals3
Anxa1
Trem2
Cd9
Atp6v0d2
Ctsl
Rgs1
Fabp5
Slc37a2
Clic4
Mmp12
Myo1e
Nceh1
Cd300c2
Atf3
Fam129b
Glipr1
Tnip3
I
Adipose Tissue vs Hepatic LAMs
-log10 p-value
log2 fold change
J
Human SAM Signature A
UMAP2
UMAP1

Figure S6: Localization of hepatic LAMs in MAFLD; related to Figure 5.

TIM4$^+$ ResKCs, TIM4$^-$ moKCs and CLEC4F$^-$ macs were sorted from mice (n=5/6 per population) fed the SD or WD for 12 (ResKCs only) or 24 weeks for Bulk RNA-seq analysis. (A) PCA plot of bulk RNA-seq data (left) and Triwise plot (right) showing DEGs between moKCs, ResKCs (SD and WD) and CLEC4F$^-$ macs from the 24 week timepoint. (B) The signature of each population used for the bulk analysis (from mice fed the WD for 24weeks) was mapped onto the liver mac UMAP to identify cells with a similar profile using the Signature Finder algorithm (Pont et al., 2019). (C) Triwise plot showing expression of DEGs from scRNA-seq analysis between the Mac1, Mac2 and moKC populations. (D) Expression of indicated genes by all monocytes and macrophages across diets and timepoints. (E) Heatmap showing top DEGs between Mac2, Mac1 and moKC populations from mice fed the WD for 24 and 36 weeks pooled (from scRNA-seq, see Fig. 5A) and their expression by the sorted populations in the bulk RNA-seq analysis. (F) *Clec4f*-DTR mice or WT littermate controls fed normal chow were given DT 3 or 6 days prior to analysis for expression of CLEC2 and CLEC4F expression. Data are representative of 2 experiments with n=4-6 per group. (G) *Nr1h3*$^{fl/fl}$ mice (no CRE; WT) were fed the WD for 24 weeks and CLEC4F, TIM4, CLEC2 and CD38 expression were examined. Left; flow cytometry plots. Right; % of total CLEC4F$^-$ macs expressing CLEC2 and CD38 (pre-moKCs) or lacking expression of both markers (Mac2). Data are from a single experiment with n=6. Error bars indicate ±SEM. (H) Heatmap showing expression of top genes associated with adipose tissue LAMs (Jaitin et al., 2019) by the indicated macrophage populations. (I) Volcano plot showing differences between adipose tissue and hepatic LAMs. (J) Signature A of the Scar-associated macrophages in human fibrotic liver (Ramachandran et al., 2019) was mapped onto the liver mac UMAP to identify cells with a similar profile using the Signature Finder algorithm (Pont et al., 2019).

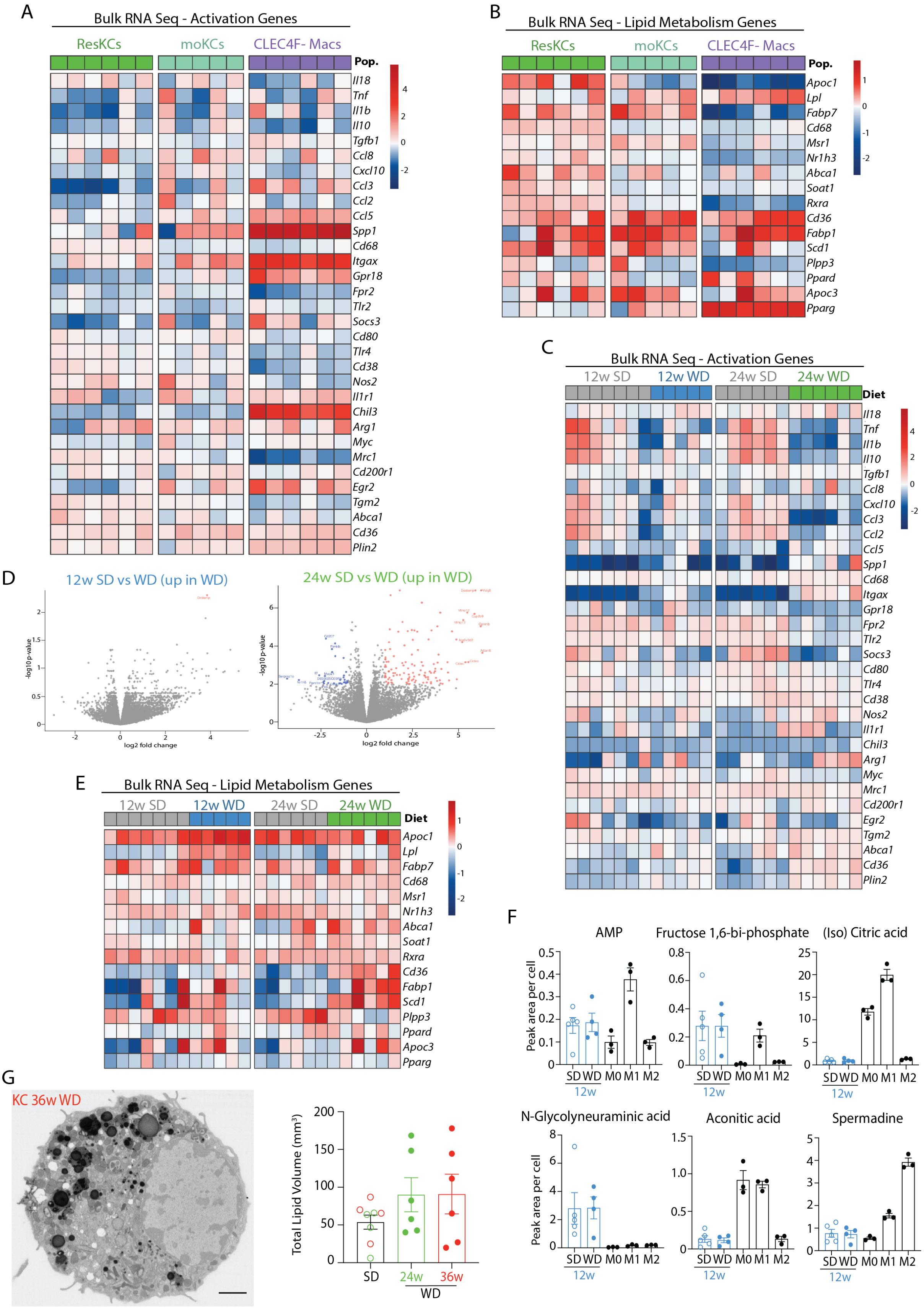

A
Bulk RNA Seq - Activation Genes
ResKCs
moKCs
CLEC4F- Macs
Pop.
Il18
Tnf
Il1b
Il10
Tgfb1
Ccl8
Cxcl10
Ccl3
Ccl2
Ccl5
Spp1
Cd68
Itgax
Gpr18
Fpr2
Tlr2
Socs3
Cd80
Tlr4
Cd38
Nos2
Il1r1
Chil3
Arg1
Myc
Mrc1
Cd200r1
Egr2
Tgm2
Abca1
Cd36
Plin2
4
2
0
-2
B
Bulk RNA Seq - Lipid Metabolism Genes
ResKCs
moKCs
CLEC4F- Macs
Pop.
Apoc1
Lpl
Fabp7
Cd68
Msr1
Nr1h3
Abca1
Soat1
Rxra
Cd36
Fabp1
Scd1
Plpp3
Ppard
Apoc3
Pparg
1
0
-1
-2
C
Bulk RNA Seq - Activation Genes
12w SD
12w WD
24w SD
24w WD
Diet
Il18
Tnf
Il1b
Il10
Tgfb1
Ccl8
Cxcl10
Ccl3
Ccl2
Ccl5
Spp1
Cd68
Itgax
Gpr18
Fpr2
Tlr2
Socs3
Cd80
Tlr4
Cd38
Nos2
Il1r1
Chil3
Arg1
Myc
Mrc1
Cd200r1
Egr2
Tgm2
Abca1
Cd36
Plin2
4
2
0
-2
D
12w SD vs WD (up in WD)
24w SD vs WD (up in WD)
-log10 p-value
log2 fold change
E
Bulk RNA Seq - Lipid Metabolism Genes
12w SD
12w WD
24w SD
24w WD
Diet
Apoc1
Lpl
Fabp7
Cd68
Msr1
Nr1h3
Abca1
Soat1
Rxra
Cd36
Fabp1
Scd1
Plpp3
Ppard
Apoc3
Pparg
1
0
-1
-2
F
AMP
Fructose 1,6-bi-phosphate
(Iso) Citric acid
N-Glycolyneuraminic acid
Aconitic acid
Spermadine
Peak area per cell
SD WD M0 M1 M2
12w
G
KC 36w WD
Total Lipid Volume (mm³)
SD
24w
36w
WD

Figure S7: Features of ResKCs in MAFLD; related to Figures 6 and 7.

(A-C) Heatmaps showing expression (measured by bulk RNA-seq) of (A,C) prototypical immune activation genes or (B) lipid metabolism associated genes by ResKCs, moKCs and CLEC4F$^-$ macs from mice fed the WD for 24 weeks (A,B) or ResKCs from mice fed either the SD or WD for 12 or 24 weeks. (D) Volcano plot showing DEGs between ResKCs fed the SD and WD for either 12 (top) or 24 (bottom) weeks. (E) Heatmap showing expression of lipid metabolism associated genes by ResKCs from mice fed the SD or WD for 12 or 24 weeks as measured by bulk RNA-seq. (F) Generation of indicated metabolites by ResKCs isolated from the livers of mice fed a SD or WD for 12 weeks, and by *in vitro* polarized M0 or M1 and M2 BM-derived macrophages. Data are from a single experiment with n=3-5 per group. Error bars indicate ±SEM. (G) CLEC4F$^+$ KCs were sorted from mice fed either the SD or the WD for 24 or 36 weeks and FIB-SEM imaging was performed. Left; image of a KC from a mouse fed the WD for 36 weeks. Right; total lipid volume (mm^3) in CLEC4F$^+$ KCs as defined by machine learning from FIB-SEM images, each point represents a cell. 2-3 cells were imaged from 2 different mice per diet and timepoint. Error bars indicate ±SEM.
